# Effect-Directed Analyses of Bioactives in Tree of Heaven (*Ailanthus altissima* (Mill.) Swingle)

**DOI:** 10.3390/plants15071026

**Published:** 2026-03-26

**Authors:** Irena Vovk, Vesna Glavnik, Simona Strgulc Krajšek, Maja Bensa, Péter G. Ott, Ágnes M. Móricz

**Affiliations:** 1 Laboratory for Food Chemistry, National Institute of Chemistry, Hajdrihova 19, SI-1000 Ljubljana, Slovenia; 2 Department of Biology, Biotechnical Faculty, University of Ljubljana, Jamnikarjeva ulica 101, SI-1000 Ljubljana, Slovenia; simona.strgulc@bf.uni-lj.si; 3 Research Institute of Faculty of Health Sciences, Faculty of Health Sciences, University of Ljubljana, Zdravstvena pot 5, SI-1000 Ljubljana, Slovenia; maja.bensa@zf.uni-lj.si; 4 Plant Protection Institute, HUN-REN Centre for Agricultural Research, Fehérvári út 132–144, 1116 Budapest, Hungary; ott.peter@atk.hun-ren.hu (P.G.O.); moricz.agnes@atk.hun-ren.hu (Á.M.M.)

**Keywords:** tree of heaven, Simaroubaceae, antimicrobial activity, enzyme inhibitor, effect-directed analyses, non-target screening, α-glucosidase, lipase, acetylcholinesterase, bioactivity profiles

## Abstract

This study evaluated activities of crude extracts from different parts of the tree of heaven (*Ailanthus altissima* (Mill.) Swingle) collected in Slovenia and Hungary, using effect-directed analyses based on hyphenation of high-performance thin-layer chromatography (HPTLC) and nine *in vitro* assays performed *in situ* on chromatographic plates after the separation. HPTLC separation combined with a set of four antibacterial assays, two antifungal assays, and three enzyme inhibitor assays to evaluate the extracts of 15 plant parts: young shoots, young leaves, mature leaves, yellow leaves, petioles of leaves, petioles of male inflorescences, petioles of fruits, female inflorescences, young fruits, male inflorescences, mature male inflorescences, bark of 1–2-year branches, bark of 2-year branches, bark of tree trunk, and bark of roots. Antibacterial activities against Gram-positive bacteria (*Bacillus subtilis*, *Rhodococcus fascians*) and Gram-negative bacteria (*Aliivibrio fischeri*, *Pseudomonas syringae* pv. *maculicola* (*Psm*)), as well as inhibition of enzymes α-glucosidase, lipase, and acetylcholinesterase, were observed for all extracts. Extracts differed in their antifungal activities. Extracts of young shoots, mature leaves, petioles of leaves, and bark of roots showed antifungal activity against plant pathogens *Fusarium avenaceum* and *Bipolaris sorokiniana.* Extracts of yellow leaves, male inflorescences, bark of 1–2-year branches, and bark of tree trunks were only active against *F. avenaceum*, whereas extracts of young leaves were only active against *B. sorokiniana*. This study is the first to report that *A. altissima* extracts exhibit (1) antifungal activity against *F. avenaceum* and *B. sorokiniana*; (2) antibacterial activity against *A. fischeri*, *Psm*, *R. fascians*, and *B. subtilis* (except leaves, bark of branches and bark of tree trunks); and (3) inhibitory activity toward lipase, α-glucosidase (except bark of tree trunks), and acetylcholinesterase (except bark of tree trunks).

## 1. Introduction

*Ailanthus altissima* (Mill.) Swingle (tree of heaven, Simaroubaceae) is native to China and North Vietnam but invasive in most other continents except Antarctica. In the 18th century, the tree was introduced to Europe as an ornamental tree, as well as for other purposes, including erosion control and as a forage for the Chinese silk-producing caterpillar *Samia cynthia* [1]. To minimize the adverse effects of alien species on biodiversity, the European Union introduced regulations, including Regulation (EU) No. 1143/2014 [2] and Commission Implementing Regulation (EU) 2019/1262 [3], where *A. altissima* is included on the list of invasive alien species of Union concern. In Europe, *A. altissima* grows widely in the warm Mediterranean region in cities and rural areas [1]. It can also be found in the lowlands and low mountain ranges from the warm southern regions of Europe (meridional zone) to the cooler central/northern regions (temperate zone), although in the north it grows primarily in urban areas due to more favorable climates [1].

*A. altissima* was described as an early-successional pioneer species with low shade tolerance, early fecundity, and large seed production [4]. Later, it was discovered that *A. altissima* could have a higher shade tolerance, as even relatively low-light conditions allowed for growth and survival of generative regeneration of the species [4]. It reproduces sexually and vegetatively, generally reaching flowering maturity after 3–5 years [1]. With increased tree heights the number of produced fruits (samaras) increases exponentially [1]. The seed germination rates are high [1], and the seeds stay viable in the soil bank for more than two years [5].

*A. altissima* is widespread in Slovenia, found most frequently in urban areas, near the Adriatic coast, on the Carst, in the Soča Valley and around Brežice in the eastern part of Slovenia [6]. The species is also widespread in Hungary and present in all regions of the country [7] (p. 239). A study in the Pannonian phytogeographical region in Hungary found that fully developed stands of *A. altissima* can outcompete and displace other plant species through intense shading and allelopathic interference, ultimately leading to reduced diversity within the canopy layer [8].

Dried bark of *A. altissima* has been used in traditional medicine in China, Korea, and India for treatment of various diseases (e.g., asthma, epilepsy, spermatorrhea, bleeding, and ophthalmic diseases) [9]. Traditional and clinical uses of different formulations (e.g., pills, tablets, decoctions, and powders) of dried bark combined with different Chinese herbs have been reported for China [9]. More than 200 compounds have been isolated from its dried bark, and many of its traditional uses have been supported by various bioassays [9]. However, further studies are required to clarify the existing gaps in its pharmacokinetic and toxicity profiles and to establish a safe therapeutic window [9].

Various bioactive secondary metabolites from different groups, like phenolic acids [10,11,12,13,14,15], flavonoids [10,11,12,13,15,16,17], stilbenoids [11], chalcones [11,16], terpenoids [14,15], monoterpenes [18], triterpenoids [14,16,19], quassinoids [19,20,21,22], sterones [14,15,19] steroids, phytosterols [15,17], phenylpropanoids [16,23], coumarins [15,16,24] and alkaloids [15,16,25,26], have been found in leaves [10,11,12,15,17,18,19,22], young leaves [20], old leaves [20], young petioles [20], flowers [12], stems [20,26], stem bark [12,14,21], bark [16,20,26], wood [20], roots [20,25], and root bark [23,24]. Several phenolic acids and flavonoids were quantified in extracts from flowers, leaves, and stem bark [12]. Phenolic acids and flavonoids contents were also determined in leaves collected in different regions [13]. Contents of phenolic acids (hydroxybenzoic and hydroxycinnamic acid derivatives), flavan-3-ols, flavonols, flavones, flavanonols, chalcones, and stilbenoids were determined in extracts from leaves [11]. Quassinoids contents were reported for extracts from young leaves, old leaves, young petioles, stems, bark, wood and roots [20].

A recently published review [27] summarized many biological activities of *A. altissima* extracts and isolated compounds, such as antibacterial [16,26], antiviral [28], antioxidant [12,16,29,30], anti-inflammatory [31,32], antimalarial [33], hypotensive [34], anticoagulant [34], smooth muscle relaxant [34], neuroprotective [16,35], DNA protective [12], anti-enzymatic [18], and cytotoxic [23,24,25] activities. Insecticidal activity (against the pea aphid, *Acyrtosiphon pisum* (Harris)), was reported for quassinoid ailanthone isolated from aqueous extracts of leaves [22]. According to an *in vitro* study, the acaricidal activity (against two tick species: *Rhipicephalus* (formerly *Boophilus*) *microplus* and *Hyalomma anatolicum*) of methanol (80%) extract from *A. altissima* leaves was superior compared to that of a commercially available acaricide permethrin used as a positive control [36]. The hydro-alcoholic (50%, *V/V*) extract of leaves modulated plant defense-related activities of chitinase and peroxidase *in vitro* [11].

*In vitro* assessment of the biological activities of *A. altissima* extracts, fractions or isolated compounds has been performed using different methodological approaches, such as disk diffusion test [10], dilution method [15], and a combination of thin-layer chromatography (TLC) and a biological assay—so-called effect-directed analysis (EDA) and TLC–EDA [26]. An example of the latter approach includes a TLC–*Bacillus subtilis* assay, which in combination with TLC-heated electrospray high-resolution tandem mass spectrometry (HESI-HRMS/MS) supported bioassay-guided isolation and identification of six antibacterial compounds from methanol extracts of *A. altissima* stem and trunk bark [26]. Chromatographic separation performed by high-performance thin-layer chromatography (HPTLC) or high-performance liquid chromatography (HPLC) can be combined with activity assays. However, combining HPTLC with a variety of activity assays [26,37,38,39,40,41,42] is much easier, faster and more cost-effective, while in the case of HPLC separation this is mostly not the case [43].

The aim of this study was to discover antimicrobial (antibacterial and antifungal) and enzyme inhibitor activities with nontargeted (HP)TLC and (HP)TLC–EDA analyses of crude extracts prepared from different parts of *A. altissima* collected in Slovenia and Hungary. Chemical and bioactivity profiles were obtained for crude extracts of young shoots, young leaves, mature leaves, yellow leaves, petioles of leaves, petioles of male inflorescences, petioles of fruits, female inflorescences, male inflorescences, mature male inflorescences, bark of 1–2-year branches, bark of 2-year branches, bark of tree trunks, and bark of roots. Activities evaluated with (HP)TLC–EDA included: (1) antibacterial activities against Gram-positive bacteria (*Bacillus subtilis*, *Rhodococcus fascians*) and Gram-negative bacteria (*Aliivibrio fischeri*, *Pseudomonas syringae* pv. *maculicola*); (2) antifungal activity against *Bipolaris sorokiniana* and *Fusarium avenaceum*; and (3) inhibitory activities against enzymes α-glucosidase, lipase, and acetylcholinesterase.

## 2. Results and Discussion

### 2.1. Extraction, Separation, Derivatization and Detection Conditions

**Figure 1:**
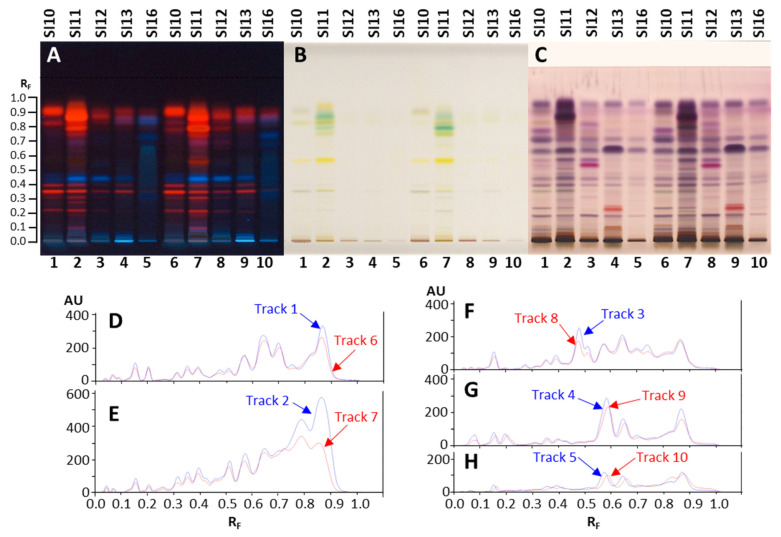
HPTLC chromatograms (**A**–**C**) and densitograms (**D**–**H**) of extracts (10 µL, 200 mg/mL) obtained from 5 selected samples by ultrasound-assisted extraction (tracks 1–5) and maceration (tracks 6–10). The HPTLC silica gel plate was developed with toluene-ethyl acetate-methanol (11:7:2, *V/V/V*) and documented after development (at 366 nm (**A**) and under white light (**B**)) and after derivatization with anisaldehyde reagent (under white light (**C**) and at 535 nm (**D**–**H**)). Applications: tracks 1 and 6: young fruits SI10; tracks 2 and 7: mature leaves SI11; tracks 3 and 8: petioles of the leaves and rachises SI12; tracks 4 and 9: bark of 2-year branches SI13; tracks 5 and 10: bark of 1–2-year branches SI6.

Chromatographic profiles obtained by HPTLC analyses of methanol extracts prepared from five samples of *A. altissima* by means of ultrasound-assisted extraction (Figure 1A–C, tracks 1–5) and maceration (Figure 1A–C, tracks 6–10) were compared visually and after densitometric scanning at 535 nm. As evident from densitograms after derivatization with anisaldehyde reagent (Figure 1D–H), the extracts prepared with ultrasound-assisted extraction (Figure 1D–H, tracks 1–5) showed higher peaks than the extracts prepared with maceration (Figure 1D–H, tracks 6–10). Therefore, ultrasound-assisted extraction was used for the preparation of extracts of all samples.

**Figure 2:**
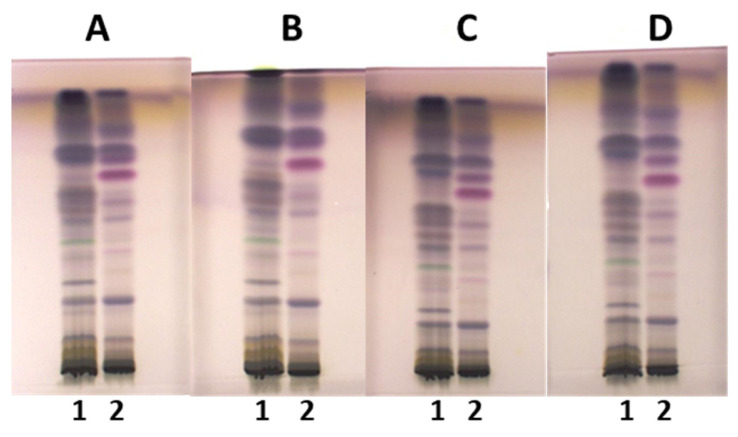
The influence of the developing solvents, toluene-ethyl acetate-methanol (5:4:1, *V/V/V*) (**A**,**B**) and toluene-isopropyl acetate-methanol (5:4:1, *V/V/V*) (**C**,**D**), and the developing distance ((**A**,**C**): 8 cm; (**B**,**D**): 9 cm) on the separation of the methanolic extracts (ultrasound-assisted extraction; 10 µL, 200 mg/mL) on the HPTLC silica gel F_254_ plates developed in a saturated (10 min) twin-trough chamber. Documentation was performed under white light after derivatization with anisaldehyde reagent. Track 1: young shoots SI2; track 2: bark from 1–2-year-old branches SI3.

**Figure 3:**
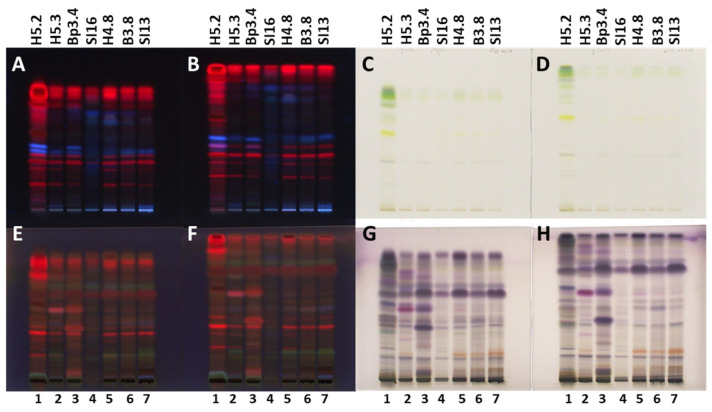
The influence of the developing chamber’s saturation on the separation and resolution in analyses of extracts (10 µL, 200 mg/mL) obtained from 7 selected samples. The HPTLC silica gel plates were developed with toluene-isopropyl acetate-methanol (5:4:1, *V/V/V*) in a twin-trough chamber saturated with filter paper (**A**,**C**,**E**,**G**) or without filter paper (**B**,**D**,**F**,**H**). Plates were documented before (**A**–**D**) and after (**E**–**H**) derivatization with anisaldehyde reagent at 366 nm (**A**,**B**,**E**,**F**) and under white light (**C**,**D**,**G**,**H**). Applications: track 1: mature leaves H5.2; track 2: petioles of leaves H5.3; track 3: male inflorescences Bp3.4; track 4: bark of 1–2-year branches SI6; track 5: bark of 1–2-year branches H4.8; track 6: bark of 1–2-year branches B3.8; track 7: bark of 2-year branches SI13.

Developing conditions for TLC and HPTLC plates were optimized for the developing solvent, the developing distance and the saturation of the twin-trough chamber. The developing solvent toluene-ethyl acetate-methanol (11:7:2, *V*/*V/V*) (Figure 1), initially used for selection of ultrasound-assisted extraction, was further optimized by changing the ratios of the solvent constituents, resulting in a mixture of toluene-ethyl acetate-methanol (5:4:1, *V/V/V*) (Figure 2A,B). Examination of the influence of the developing distance (8 and 9 cm) was performed using two developing solvents, toluene-ethyl acetate-methanol (5:4:1, *V/V/V*) (Figure 2A,B) and toluene-isopropyl acetate-methanol (5:4:1, *V/V/V*) (Figure 2C,D), that differed only in one constituent being either ethyl acetate or isopropyl acetate. As evident from Figure 2, the best resolution among the chromatographic zones in the tracks of extracts of young shoots SI2 (Figure 2, track 1) and bark from 1–2 years old branches SI3 (Figure 2, track 2) was achieved using toluene-isopropyl acetate-methanol (5:4:1, *V/V/V*) and a developing distance of 9 cm (Figure 2D). For this reason, these conditions were applied for all further analyses. As expected, the use of filter paper for the saturation of the developing chamber resulted in lower R_F_ values and, in some cases, in lower resolution between the chromatographic zones compared to the “saturation” without the filter paper (Figure 3). Therefore, further analyses were performed without using filter paper.

**Figure 4:**
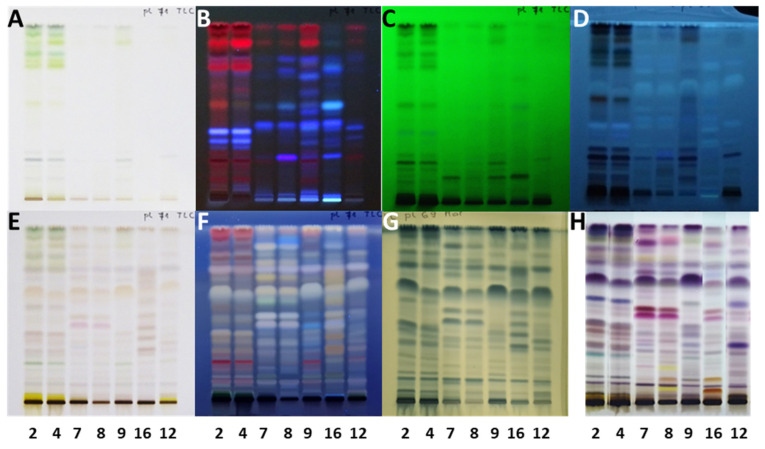
HPTLC analyses of 7 extracts (10 µL, 200 mg/mL) on the HPTLC silica gel F_254_ plates developed with toluene-isopropyl acetate-methanol (5:4:1, *V/V/V*) and documented under white light (**A**,**E**,**G**,**H**) and at 366 nm (**B**,**D**,**F**) and 254 nm (**C**) before (**A**–**C**) and after application of detection reagents (10% sulfuric acid (**E**,**F**), primuline (**D**), molybdatophosphoric acid (**G**), and anisaldehyde (**H**)). Track 2: young leaves H2.2; track 4: mature leaves Bp5.2; track 7: petioles of leaves H7.3; track 8: young shoots Bp2.10; track 9: bark of 1–2-year branches SI6; track 12: male inflorescences Bp3.4; track 16: petioles of fruits Bp5.15.

A big advantage of the (HP)TLC that enables detection of all compounds in any sample after development and/or after post-chromatographic derivatization in either absorption or fluorescence mode was used to obtain complementary chemical profiles. As shown in Figure 4, eight different chemical profiles were obtained by comparisons of chromatograms of each of the seven selected samples documented after development under white light (A,E,G,H) at 254 nm (C) and 366 nm (B,D,F) before (A,B,C) and after application of detection reagents (10% sulfuric acid (E,F), primuline (D), molybdatophosphoric acid (G), and anisaldehyde (H)) (Figure 4). Based on the chemical profiles, 19 samples were selected for HPTLC and effect-directed analyses focused on detection of antibacterial, antifungal and enzyme inhibitor activities of the extracts.

### 2.2. TLC and HPTLC–Antibacterial Bioassays of 19 Selected Extracts

#### 2.2.1. TLC–*Aliivibrio fischeri* Bioassay

**Figure 5:**
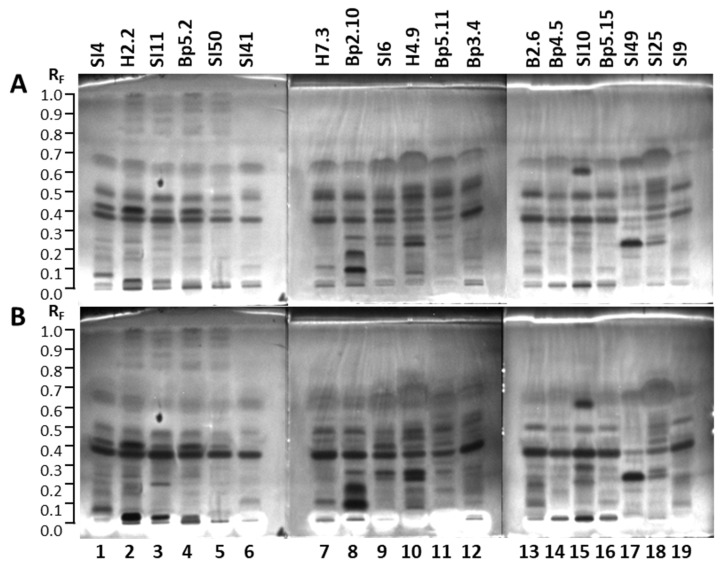
TLC–*Aliivibrio fischeri* bioluminescent bioautograms of 19 selected extracts (5 µL, 200 mg/mL) on the HPTLC silica gel F_254_ plates developed with toluene-isopropyl acetate-methanol (5:4:1, *V/V/V*). Bioluminescent bioautograms were documented instantly (**A**) and 60 min (**B**) after the bioassay. Track 1: young leaves SI4; track 2: young leaves H2.2; track 3: mature leaves SI11; track 4: mature leaves Bp5.2; track 5: yellow leaves SI50; track 6: petioles of leaves SI41; track 7: petioles of leaves H7.3; track 8: young shoots Bp2.10; track 9: bark of 1–2-year branches SI6; track 10: bark of tree trunk H4.9; track 11: bark of roots Bp5.11; track 12: male inflorescences Bp3.4; track 13: petioles of male inflorescences B2.6; track 14: female inflorescences Bp4.5; track 15: young fruits SI10; track 16: petioles of fruits Bp5.15; track 17: bark of tree trunk SI49; track 18: bark of 2-year branches SI25; track 19: mature male inflorescences SI9.

Examination of the activity of the 19 extracts against Gram-negative bacteria performed by TLC–*Aliivibrio fischeri* bioassays (Figure 5) resulted in several dark zones on a bright bioluminescent background confirming antibacterial activity in all the extracts instantly (Figure 5A), as well as 60 min after the bioassay (Figure 5B). The results are summarized in Table 1. Comparison of the intensities of dark zones observed instantly (Figure 5A) and 60 min (Figure 5B) after the bioassay confirmed higher intensities of almost all dark zones for all 19 extracts 60 min after the bioassay, indicating strong, long-lasting antibacterial activity. The only exceptions were dark zones observed at the start position or close to the start position (R_F_ 0.02 and 0.03) in tracks of all 19 extracts instantly after the bioassay (Figure 5A). Only for the extracts of young leaves H2.2 (track 2), mature leaves SI11 (track 3) and Bp5.2 (track 4), female inflorescences Bp4.5 (track 14), young fruits SI10 (track 15), and petioles of fruits Bp5.15 (track 16) were one or two dark zones at the start position or close to the start position more intense 60 min after the bioassay (Figure 5B) than instantly (Figure 5A), indicating strong, long-lasting antibacterial activity. The most intense among these zones was observed at R_F_ 0.03 for the extract of young leaves H2.2 (track 2) 60 min after the bioassay (Figure 5B). In most other cases, these dark zones at the start position or close to the start position became less intense or could not be detected 60 min after the bioassay due to the very intense white zones around them (Figure 5B) that were not observed instantly after the bioassay (Figure 5A).

Dark zones at the start position or close to the start position were not detected 60 min after the bioassay for extracts of bark of tree trunk H4.9 (track 10), bark of roots Bp5.11 (track 11), bark of tree trunk SI49 (track 17), and mature male inflorescences SI9 (track 19) (Figure 5B). One of the two zones detected at the start position or close to the start position instantly after the bioassay (Figure 5A) could not be detected 60 min after the bioassay (Figure 5B) for the following extracts: young leaves SI4 (track 1), yellow leaves SI50 (track 5), petioles of leaves SI41 (track 6), petioles of leaves H7.3 (track 7), young shoots Bp2.10 (track 8), bark of 1–2-year branches SI6 (track 9), petioles of male inflorescences B2.6 (track 13), and petioles of fruits Bp5.15 (track 16). Dark zones at the start position or close to the start position were less intense 60 min after the bioassay for extracts of petioles of leaves SI41 (track 6), bark of 1–2-year branches SI6 (track 9), and male inflorescences Bp3.4 (track 12) (Figure 5B).

**Table 1:** Activities of 19 *A. altissima* extracts against Gram-negative bacteria (*Aliivibrio fischeri*, *Pseudomonas syringae* pv. *maculicola*); Gram-positive bacteria *(Bacillus subtilis*, *Rhodococcus fascians*), fungal plant pathogens *(Bipolaris sorokiniana*, *Fusarium avenaceum*); and enzymes α-glucosidase, lipase, and acetylcholinesterase.

No.	Code	Plant Parts	*Aliivibrio fischeri*	*Bacillus subtilis*	*Rhodococcus fascians*	*Pseudomonas* *syringae* pv. *maculicola*	*Bipolaris sorokiniana*	*Fusarium avenaceum*	α-Glucosidase	Lipase	Acetylcholinesterase
9	SI6	Bark of 1–2-year branches	+	+	+	+	-	+	+	+	+
18	SI25	Bark of 2-year branches	+	+	+	+	-	-	+	+	+
11	Bp5.11	Bark of roots	+	+	+	+	+	+	+	+	+
17	SI49	Bark of tree trunk	+	+	+	+	-	-	+	+	+
10	H4.9	Bark of tree trunk	+	+	+	+	-	+	+	+	+
14	Bp4.5	Female inflorescences	+	+	+	+	-	-	+	+	+
12	Bp3.4	Male inflorescences	+	+	+	+	-	+	+	+	+
3	SI11	Mature leaves	+	+	+	+	-	+	+	+	+
4	Bp5.2	Mature leaves	+	+	+	+	+	-	+	+	+
19	SI9	Mature male inflorescences	+	+	+	+	-	-	+	+	+
6	SI41	Petioles of leaves	+	+	+	+	-	+	+	+	+
7	H7.3	Petioles of leaves	+	+	+	+	+	+	+	+	+
13	B2.6	Petioles of male inflorescences	+	+	+	+	-	-	+	+	+
16	Bp5.15	Petioles of fruits	+	+	+	+	-	-	+	+	+
5	SI50	Yellow leaves	+	+	+	+	-	+	+	+	+
15	SI10	Young fruits	+	+	+	+	-	-	+	+	+
1	SI4	Young leaves	+	+	+	+	+	-	+	+	+
2	H2.2	Young leaves	+	+	+	+	+	-	+	+	+
8	Bp2.10	Young shoots	+	+	+	+	+	+	+	+	+

+ Activity detected; - No activity detected.

As evident from Figure 5, dark zones at R_F_ 0.36 were observed for all extracts instantly (Figure 5A) and 60 min (Figure 5B) after the bioassay, and all these zones were more intense after 60 min. The lowest intensity among the dark zones at this R_F_ was detected for the extract of bark of tree trunk SI49 (track 17) (Figure 5A,B), while the highest intensities were detected for the extracts of mature leaves SI11 (track 3), petioles of leaves H7.3 (track 7), young shoots Bp2.10 (track 8), petioles of male inflorescences B2.6 (track 13), and young fruits SI10 (track 15) (Figure 5A,B). These dark zones at R_F_ 0.36 were also the most intense zones both instantly after the bioassay (Figure 5A) and 60 min later (Figure 5B) for most of the 19 extracts, except for the extracts of young leaves H2.2 (track 2), young shoots Bp2.10 (track 8), and bark of tree trunk SI49 (track 17). The extract of young leaves H2.2 (track 2) exhibited the most intense dark zone at R_F_ 0.41 instantly after the bioassay (Figure 5A), while 60 min later, dark zones at R_F_ 0.36 and R_F_ 0.41 displayed comparable intensities (Figure 5B). The extract of young shoots Bp2.10 (track 8) showed the most intense dark zone at R_F_ 0.09 instantly after the bioassay (Figure 5A), while 60 min later, dark zones at R_F_ 0.09 and R_F_ 0.18 showed comparable intensities and were more intense than the zone at R_F_ 0.36 (Figure 5B). The extract of bark of tree trunk SI49 (track 17) showed the most intense zone at R_F_ 0.23 both instantly after the bioassay (Figure 5A) and 60 min later (Figure 5B).

Dark zones at R_F_ 0.47 were observed for all 19 extracts, and their intensity was slightly higher instantly (Figure 5A) than 60 min after the bioassay (Figure 5B). The extract of petioles of male inflorescences B2.6 (track 13) showed the highest intensity, and the extract of bark of tree trunk SI49 showed the lowest intensity of the zone at R_F_ 0.47 (track 17) (Figure 5). An intense dark zone at R_F_ 0.61 was only detected for the extract of young fruits SI10 (track 15) and was more intense 60 min after the bioassay (Figure 5B) than instantly (Figure 5A).

The highest number of antibacterial dark zones that were spread from the start to R_F_ 0.97 was found for the extract of young leaves H2.2 (track 2) (Figure 5). Antibacterial dark zones in the R_F_ region between 0.80 and 0.97 were detected only in the following extracts: young leaves SI4 (track 1) and H2.2 (track 2); mature leaves SI11 (track 3) and Bp5.2 (track 4); yellow leaves SI50 (track 5), bark of 1–2-year branches SI6 (track 9), and young fruits SI10 (track 15) (Figure 5). Among these extracts young leaves H2.2 (track 2), mature leaves SI11 (track 3) and Bp5.2 (track 4), and yellow leaves SI50 (track 5) had the highest number and the most intense dark zones in the R_F_ region between 0.80 and 0.97 (Figure 5).

Extracts of young shoots Bp2.10 (track 8), bark of tree trunk H4.9 (track 10), and young fruits SI10 (track 15) (Figure 5A,B) exhibited higher numbers of intense antibacterial dark zones than other extracts instantly after the bioassay (Figure 5A) and 60 min later (Figure 5B).

There is no report in the literature related to the activity of *A. altissima* extracts or fractions against *Aliivibrio fischeri*. To the best of our knowledge, this is the first report on the inhibition of *Aliivibrio fischeri* growth by *A. altissima* extracts of young shoots, young leaves, mature leaves, yellow leaves, petioles of leaves, petioles of male inflorescences, petioles of fruits, female inflorescences, male inflorescences, mature male inflorescences, young fruits, bark of 1–2-year branches, bark of 2-year branches, bark of tree trunks, and bark of roots.

#### 2.2.2. TLC–*Bacillus subtilis* Bioassay

**Figure 6:**
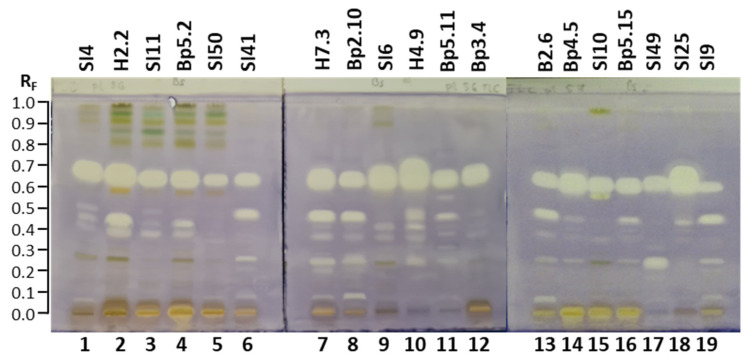
TLC–*Bacillus subtilis* bioautograms of 19 selected extracts (10 µL, 200 mg/mL) on the HPTLC silica gel F_254_ plates developed with toluene-isopropyl acetate-methanol (5:4:1, *V/V/V*) documented under white light instantly after the bioassay. Track 1: young leaves SI4; track 2: young leaves H2.2; track 3: mature leaves SI11; track 4: mature leaves Bp5.2; track 5: yellow leaves SI50; track 6: petioles of leaves SI41; track 7: petioles of leaves H7.3; track 8: young shoots Bp2.10; track 9: bark of 1–2-year branches SI6; track 10: bark of tree trunk H4.9; track 11: bark of roots Bp5.11; track 12: male inflorescences Bp3.4; track 13: petioles of male inflorescences B2.6; track 14: female inflorescences Bp4.5; track 15: young fruits SI10; track 16: petioles of fruits Bp5.15; track 17: bark of tree trunk SI49; track 18: bark of 2-year branches SI25; track 19: mature male inflorescences SI9.

Examination of the activity of the 19 extracts against Gram-positive bacteria *Bacillus subtilis* performed by TLC–*Bacillus subtilis* bioassays instantly resulted in several bright chromatographic zones in tracks of all 19 extracts (Figure 6), indicating antibacterial activity. The results are summarized in Table 1. Regardless of the sample type used for extraction, the most intense zones showing activity against *Bacillus subtilis* were observed in the R_F_ range of 0.57 to 0.71 (Figure 6). As shown in Figure 6, in this R_F_ range the most pronounced zones were detected for bark of tree trunk H4.9 (track 10, R_F_ *≈* 0.57*–*0.71), followed by bark of 2-year branches SI25 (track 18, R_F_ *≈* 0.58*–*0.71), followed by bark of 1–2-year branches SI6 (track 9, R_F_ *≈* 0.57*–*0.68). Slightly lower but comparable intensities of zones showing antibacterial activity in the same R_F_ range (Figure 6) were observed for extracts of female inflorescences Bp4.5 (track 14 R_F_ ≈ 0.57–0.67), young leaves H2.2 (track 2, R_F_ *≈* 0.58–0.69) and SI4 (track 1, R_F_ ≈ 0.62–0.70), petioles of leaves H7.3 (track 7, R_F_ ≈ 0.58–0.66) and male inflorescences Bp3.4 (track 12, R_F_ ≈ 0.58–0.66). Even lower, but comparable intensities of the zones were obtained for extracts of mature leaves SI11 (track 3, R_F_ ≈ 0.58–0.66) and Bp5.2 (track 4, R_F_ ≈ 0.58–0.65) and bark of roots Bp5.11 (track 11, R_F_ ≈ 0.57–0.65) (Figure 6). Comparable, but slightly lower intensities of zones were detected for extracts of young shoots Bp2.10 (track 8, R_F_ ≈ 0.58–0.64), young fruits SI10 (track 15, R_F_ ≈ 0.60–0.66), petioles of male inflorescences B2.6 (track 13, R_F_ ≈ 0.60–0.67), petioles of the fruits Bp5.15 (track 16, R_F_ ≈ 0.57–0.65) and bark of tree trunk SI49 (track 17, R_F_ ≈ 0.58–0.65) (Figure 6). 

Zones for extracts of yellow leaves SI50 (track 5, R_F_ ≈ 0.58–0.64) and petioles of leaves SI41 (track 6, R_F_ ≈ 0.58–0.64) showed comparable intensities, however, their intensities were lower than the intensities of the zones of the extracts mentioned so far in the same R_F_ range (R_F_ *≈* 0.57*–*0.71) (Figure 6). Among all extracts, the zone with the lowest intensity in this R_F_ range was obtained for the extract of mature male inflorescences SI9 (Figure 6, track 19, R_F_ ≈ 0.58–0.63). However, this zone displayed intensity comparable to that of other intense zones detected at R_F_ ≈ 0.46 (Figure 6) for extracts of petioles of leaves SI41 (track 6), petioles of leaves H7.3 (track 7), young shoots Bp2.10 (track 8), bark of tree trunk H4.9 (track 10), petioles of male inflorescences B2.6 (track 13), and mature male inflorescences SI9 (track 19), as well as zones detected at R_F_ ≈ 0.42 for extract of young leaves H2.2 (track 2) and at R_F_ ≈ 0.23 for extract of bark of tree trunk SI49 (track 17) (Figure 6).

**Figure A1:**
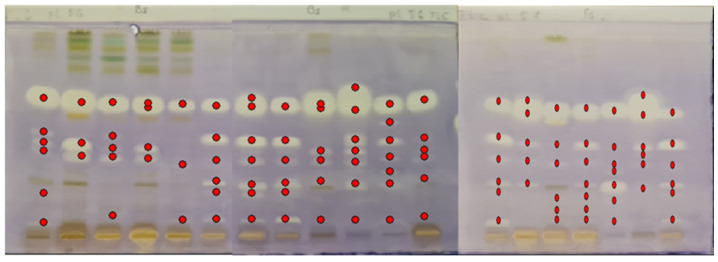
Image analysis of TLC–*B. subtilis* bioautograms of 19 selected extracts (10 µL, 200 mg/mL) on the HPTLC silica gel F_254_ plates developed with toluene-isopropyl acetate-methanol (5:4:1, *V/V/V*) documented under white light. Red circles on the zones were obtained by image analysis with SORBFIL Visualizer TLC Quantitative Evaluation software using the “Tracks/Bright spots” option. Tracks from the left to the right: track 1: young leaves SI4; track 2: young leaves H2.2; track 3: mature leaves SI11; track 4: mature leaves Bp5.2; track 5: yellow leaves SI50; track 6: petioles of leaves SI41; track 7: petioles of leaves H7.3; track 8: young shoots Bp2.10; track 9: bark of 1–2-year branches SI6; track 10: bark of tree trunk H4.9; track 11: bark of roots Bp5.11; track 12: male inflorescences Bp3.4; track 13: petioles of male inflorescences B2.6; track 14: female inflorescences Bp4.5; track 15: young fruits SI10; track 16: petioles of fruits Bp5.15; track 17: bark of tree trunk SI49; track 18: bark of 2-year branches SI25; track 19: mature male inflorescences SI9.

As shown in Appendix A (Figure A1), all antimicrobial zones that were detected by visual inspection were also found by image analysis performed with SORBFIL Visualizer TLC Quantitative Evaluation software using the “Tracks/Bright spots” option. Image analysis with SORBFIL software confirmed the suspicion that two zones formed a single antimicrobial zone within the R_F_ range of 0.57–0.71 in tracks of six extracts. As evident in Appendix A (Figure A1), this pattern of two zones was observed for the following extracts: mature leaves Bp5.2 (track 4), petioles of leaves H7.3 (track 7), bark of 1–2-year branches SI6 (track 9), bark of tree trunk H4.9 (track 10), female inflorescences Bp4.5 (track 14) and bark of 2-year branches SI25 (track 18). Summing peak areas of the two zones for each of the extracts in tracks 4, 7, 9, 10, 14, and 18 and comparing them with peak areas of antibacterial zones across the R_F_ range of 0.57–0.71 showed the highest total peak area for bark of the tree trunk H4.9 (track 10) and the lowest for the extract of mature male inflorescences SI9 (track 19).

In our previous study, a TLC–*Bacillus subtilis* assay and TLC–heated electrospray high-resolution tandem mass spectrometry (HESI-HRMS/MS) supported the bioassay-guided isolation and identification of six antibacterial compounds ((9Z,11E)-13-hydroxy-9,11-octadecadienoic acid (13-HODE), (10E,12Z)-9-hydroxy-10,12-octadecadienoic acid (9-HODE), hexadecanedioic acid (thapsic acid), 16-hydroxyhexadecanoic acid (juniperic acid), 16-feruloyloxypalmitic acid (alpinagalanate), and canthin-6-one) from the methanol extracts of *A. altissima* stem bark from young branches and trunk bark [26]. As shown by *in vitro* agar disk diffusion tests, crude methanol extract and its ethyl acetate, as well as butanol subfractions prepared from *A. altissima* leaves, significantly inhibited *Bacillus subtilis* ATCC 6633 growth [30]. Using a disk diffusion test, antimicrobial activity against *Bacillus subtilis* was confirmed for methanolic extracts of *A. altissima* leaves collected at different locations, but the activity was not confirmed for the hydrodistilled residues [10].

To the best of our knowledge, this is the first report on the antibacterial activity of *A. altissima* extracts of young shoots, young leaves (also in comparison with mature leaves), yellow leaves, petioles of leaves, petioles of male inflorescences, petioles of fruits, female inflorescences, male inflorescences, mature male inflorescences, young fruits, and bark of roots against *Bacillus subtilis*.

#### 2.2.3. TLC–*Rhodococcus fascians* Bioassay

**Figure 7:**
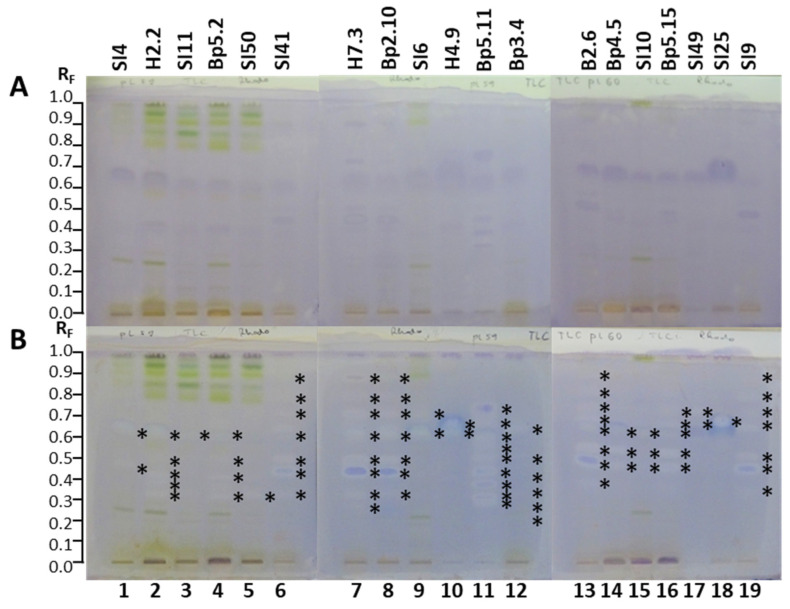
TLC–*Rhodococcus fascians* bioautograms of 19 selected extracts (10 µL, 200 mg/mL) on the TLC silica gel F_254_ plates developed with toluene-isopropyl acetate-methanol (5:4:1, *V/V/V*) documented under white light instantly after the bioassay (**A**) and 16 h after the bioassay (**B**). Track 1: young leaves SI4; track 2: young leaves H2.2; track 3: mature leaves SI11; track 4: mature leaves Bp5.2; track 5: yellow leaves SI50; track 6: petioles of leaves SI41; track 7: petioles of leaves H7.3; track 8: young shoots Bp2.10; track 9: bark of 1–2-year branches SI6; track 10: bark of tree trunk H4.9; track 11: bark of roots Bp5.11; track 12: male inflorescences Bp3.4; track 13: petioles of male inflorescences B2.6; track 14: female inflorescences Bp4.5; track 15: young fruits SI10; track 16: petioles of fruits Bp5.15; track 17: bark of tree trunk SI49; track 18: bark of 2-year branches SI25; track 19: mature male inflorescences SI9. Active zones are marked with an asterisk (*).

The TLC–*Rhodococcus fascians* bioassay of the 19 extracts resulted in several white zones, confirming the antimicrobial activity against Gram-positive bacterial phytopathogen *Rhodococcus fascians*. The results are summarized in Table 1. White zones on a bluish background were detected in tracks of all 19 extracts 16 h after the bioassay (Figure 7B) but not instantly after the bioassay (Figure 7A). However, the numbers and intensity of these white zones were quite diverse. The highest number of white zones was observed for the extract of bark of roots Bp5.11 (track 11, R_F_ ≈ 0.28, 0.31, 0.36, 0.43, 0.49, 0.55, 0.58, 0.60, 0.66 and 0.72). Only one white zone (a quite intense zone at R_F_ ≈ 0.66) was detected for the extract of bark of 2-year branches SI25 (track 18) (Figure 7). The extracts of petioles of leaves SI41 (track 6) and H7.3 (track 7), and young shoots Bp2.10 (track 8) showed the same pattern of the white zones (at R_F_ ≈ 0.31, 0.43, 0.49, 0.60, 0.70, 0.78 and 0.87), among which zones at R_F_ 0.49, 0.60 and 0.70 had the highest intensities (Figure 7). The lowest intensities of white zones were detected for extracts of mature leaves SI11 (track 3), yellow leaves SI50 (track 5), young fruits SI10 (track 15), and bark of tree trunk SI49 (track 17) (Figure 7). All white zones of these four extracts were less intense than the only white antimicrobial zone in the extract of bark of 2-year branches SI25 (track 18), both white zones in the extract of bark of tree trunk H4.9 (track 10), and the two more intense white zones in the extract of bark of 1–2-year branches SI6 (track 9) (Figure 7).

There is no report in the literature related to the activity of *A. altissima* extracts or fractions against *Rhodococcus fascians*. To the best of our knowledge, this is the first report on the antibacterial activity of *A. altissima* extracts of young shoots, young leaves, mature leaves, yellow leaves, petioles of leaves, petioles of male inflorescences, petioles of fruits, female inflorescences, male inflorescences, mature male inflorescences, young fruits, bark of 1–2-year branches, bark of 2-year branches, bark of tree trunks, and bark of roots against *Rhodococcus fascians*.

#### 2.2.4. HPTLC–*Pseudomonas syringae* pv. *maculicola* Bioassay

**Figure 8:**
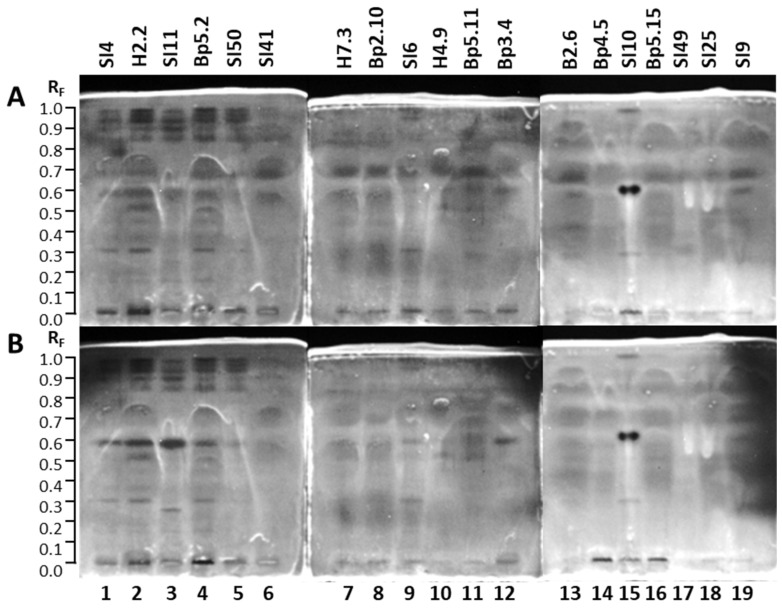
HPTLC–*Pseudomonas syringae* pv. *maculicola* bioluminescent bioautograms of 19 selected extracts (10 µL, 200 mg/mL) on the HPTLC silica gel F_254_ plates developed with toluene-isopropyl acetate-methanol (5:4:1, *V/V/V*). Plates were documented instantly (**A**) and 80 min (**B**) after the bioassay. Track 1: young leaves SI4; track 2: young leaves H2.2; track 3: mature leaves SI11; track 4: mature leaves Bp5.2; track 5: yellow leaves SI50; track 6: petioles of leaves SI41; track 7: petioles of leaves H7.3; track 8: young shoots Bp2.10; track 9: bark of 1–2-year branches SI6; track 10: bark of tree trunk H4.9; track 11: bark of roots Bp5.11; track 12: male inflorescences Bp3.4; track 13: petioles of male inflorescences B2.6; track 14: female inflorescences Bp4.5; track 15: young fruits SI10; track 16: petioles of fruits Bp5.15; track 17: bark of tree trunk SI49; track 18: bark of 2-year branches SI25; track 19: mature male inflorescences SI9.

The HPTLC–*Pseudomonas syringae* pv. *maculicola* bioassay of the 19 extracts resulted in several dark zones on a bright bioluminescent background (Figure 8) in tracks of all 19 extracts, confirming the antimicrobial activity against a Gram-negative bacterial plant pathogen *Pseudomonas syringae* pv. *maculicola*. The results are summarized in Table 1. Most of the dark zones were more intense instantly (Figure 8A) than 60 min after the bioassay (Figure 8B). The exceptions were dark zones found at the start position, as well as at R_F_ values 0.30 and 0.59, that were more intense 60 min after the bioassay than instantly (Figure 8). These more intense zones at the start position were found after 60 min for mature leaves Bp5.2 (track 4), female inflorescences Bp4.5 (track 14), and petioles of fruits Bp5.15 (track 16) (Figure 8B). Yet another more intense dark zone was detected at R_F_ 0.30 for the extract of mature leaves SI11 (track 3) 60 min after the bioassay (Figure 8B). More intense dark zones at R_F_ 0.59 were observed after 60 min for the extracts of young leaves SI4 (track 1) and H2.2 (track 2), mature leaves SI11 (track 3) and Bp5.2 (track 4), and male inflorescences Bp3.4 (track 12) (Figure 8B).

There is only one report in the literature related to the activity of *A. altissima* against *Pseudomonas syringae* pv. *maculicola*, in which the inhibitory activity of ailanstigol A and ailanstigol B (steroidal compounds isolated from ethyl acetate subfraction of methanol extract of leaves) was confirmed using a double dilution method [15]. No study has reported the activity of *A. altissima* extracts or fractions against *Pseudomonas syringae* pv. *maculicola*.

To the best of our knowledge, this is the first report on the antibacterial activity of *A. altissima* extracts of young shoots, young leaves, mature leaves, yellow leaves, petioles of leaves, petioles of male inflorescences, petioles of fruits, female inflorescences, male inflorescences, mature male inflorescences, young fruits, bark of 1–2-year branches, bark of 2-year branches, bark of tree trunks, and bark of roots against *Pseudomonas syringae* pv. *maculicola*.

### 2.3. TLC and HPTLC–Antifungal Bioassays of 19 Selected Extracts

#### 2.3.1. HPTLC–*Bipolaris sorokiniana* Bioassay

**Figure 9:**
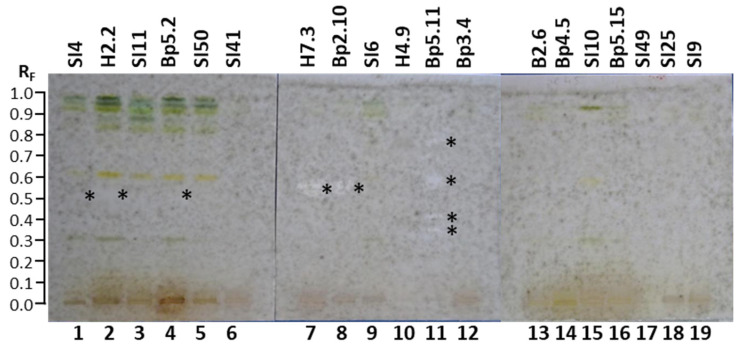
TLC–*Bipolaris sorokiniana* bioautograms of 19 selected extracts (20 µL, 200 mg/mL) on the HPTLC silica gel F254 plates developed with toluene-isopropyl acetate-methanol (5:4:1, *V/V/V*) documented under white light 52 h after the bioassay. Track 1: young leaves SI4; track 2: young leaves H2.2; track 3: mature leaves SI11; track 4: mature leaves Bp5.2; track 5: yellow leaves SI50; track 6: petioles of leaves SI41; track 7: petioles of leaves H7.3; track 8: young shoots Bp2.10; track 9: bark of 1–2-year branches SI6; track 10: bark of tree trunk H4.9; track 11: bark of roots Bp5.11; track 12: male inflorescences Bp3.4; track 13: petioles of male inflorescences B2.6; track 14: female inflorescences Bp4.5; track 15: young fruits SI10; track 16: petioles of fruits Bp5.15; track 17: bark of tree trunk SI49; track 18: bark of 2-year branches SI25; track 19: mature male inflorescences SI9. Active zones are marked with an asterisk (*).

The HPTLC–*Bipolaris sorokiniana* bioassay was used to examine the antifungal activity of all 19 extracts against *Bipolaris sorokiniana*—a fungal pathogen that causes diseases in various plants. Fifty-two hours after the bioassay, some “white” zones with a lack of visible dark gray *Bipolaris sorokiniana* hyphae indicated the inhibition zones (Figure 9). The results are summarized in Table 1. The most intense white active zones were detected at R_F_ ≈ 0.54 for the extracts of petioles of leaves H7.3 (Figure 9, track 7) and young shoots Bp2.10 (Figure 9, track 8). The highest number of white active zones (at R_F_ ≈ 0.35, 0.41, 0.58 and 0.77) was detected for the extract of bark of roots Bp5.11 (Figure 9, track 11). 

Additional active zones were observed at R_F_ ≈ 0.52 (Figure 9) for extracts of young leaves SI4 (track 1) and H2.2 (track 2), as well as mature leaves Bp5.2 (track 4). No antifungal activity against *Bipolaris sorokiniana* was detected for the following 13 extracts (Figure 9): mature leaves SI11 (track 3), yellow leaves SI50 (track 5), petioles of leaves SI41 (track 6), bark of 1–2-year branches SI6 (track 9), bark of tree trunk H4.9 (track 10), male inflorescences Bp3.4 (track 12), petioles of male inflorescences B2.6 (track 13), female inflorescences Bp4.5 (track 14), young fruits SI10 (track 15), petioles of the fruits Bp5.15 (track 16), bark of tree trunk SI49 (track 17), bark of 2-year branches SI25 (track 18), and mature male inflorescences SI9 (track 19).

There is no report in the literature about the activity of *A. altissima* extracts or fractions against *Bipolaris sorokiniana*. To the best of our knowledge, this is the first report on the inhibition activity of *A. altissima* extracts of young shoots, young leaves, mature leaves, petioles of leaves, and bark of roots against *Bipolaris sorokiniana*.

#### 2.3.2. HPTLC–*Fusarium avenaceum* Bioassay

**Figure 10:**
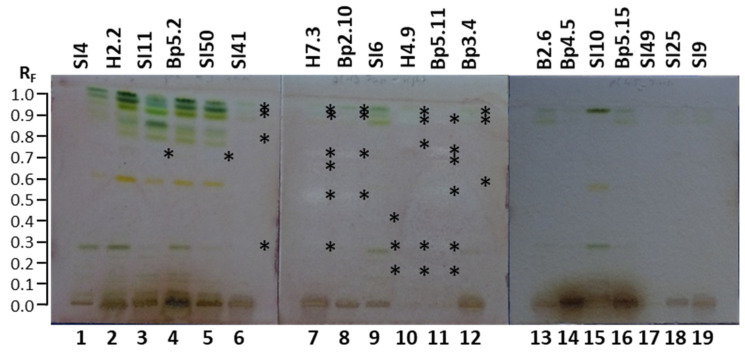
TLC–*Fusarium avenaceum* bioautograms of 19 selected extracts (20 µL, 200 mg/mL) on the HPTLC silica gel F_254_ plates developed with toluene-isopropyl acetate-methanol (5:4:1, *V/V/V*) and documented under white light. Track 1: young leaves SI4; track 2: young leaves H2.2; track 3: mature leaves SI11; track 4: mature leaves Bp5.2; track 5: yellow leaves SI50; track 6: petioles of leaves SI41; track 7: petioles of leaves H7.3; track 8: young shoots Bp2.10; track 9: bark of 1–2-year branches SI6; track 10: bark of tree trunk H4.9; track 11: bark of roots Bp5.11; track 12: male inflorescences Bp3.4; track 13: petioles of male inflorescences B2.6; track 14: female inflorescences Bp4.5; track 15: young fruits SI10; track 16: petioles of fruits Bp5.15; track 17: bark of tree trunk SI49; track 18: bark of 2-year branches SI25; track 19: mature male inflorescences SI9. Active zones are marked with an asterisk (*).

The HPTLC–*Fusarium avenaceum* bioassay was used to test the antifungal activity of all 19 extracts against *Fusarium avenaceum*—a fungal plant pathogen. Antifungal activity was detected as zones with a lack of visible *Fusarium avenaceum* hyphae. The results are summarized in Table 1. As shown in Figure 10, antifungal activity was detected at several R_F_ values in tracks of the following nine extracts: mature leaves SI11 (track 3, R_F_ ≈ 0.73), yellow leaves SI50 (track 5, R_F_ ≈ 0.73), petioles of leaves SI41 (track 6, R_F_ ≈ 0.29, 0.79, 0.91 and 0.94), petioles of leaves H7.3 (track 7, R_F_ ≈ 0.29, 0.52, 0.67, 0.73, 0.91 and 0.93), young shoots Bp2.10 (track 8, R_F_ ≈ 0.52, 0.73, 0.91 and 0.93), bark of 1–2-year branches SI6 (track 9, R_F_ ≈ 0.16, 0. 29 and 0.42), bark of tree trunk H4.9 (track 10, R_F_ ≈ 0.16, 0.29, 0.77, 0.89 and 0.92), bark of roots Bp5.11 (track 11, R_F_ ≈ 0.16, 0.29, 0.54, 0.69, 0.74 and 0.89), and male inflorescences Bp3.4 (track 12, R_F_ ≈ 0.59, 0.89 and 0.92). 

The most intense zone showing the highest antifungal activity against *Fusarium avenaceum* was observed at R_F_ ≈ 0.52 for the extract of petioles of leaves H7.3 (Figure 10, track 7). Slightly lower intensities were detected for the zones at R_F_ ≈ 0.73 for extracts of petioles of leaves H7.3 (track 7) and young shoots Bp2.10 (track 8) and the zone at R_F_ ≈ 0.74 for bark of roots Bp5.11 (track 11) (Figure 10). No antifungal activity against *Fusarium avenaceum* was detected for the following 13 extracts (Figure 10): young leaves SI4 (track 1) and H2.2 (track 2), mature leaves Bp5.2 (track 4), petioles of male inflorescences B2.6 (track 13), female inflorescences Bp4.5 (track 14), young fruits SI10 (track 15), petioles of the fruits Bp5.15 (track 16), bark of tree trunk SI49 (track 17), bark of 2-year branches SI25 (track 18), and mature male inflorescences SI9 (track 19).

There is no report in the literature about the activity of *A. altissima* extracts or fractions against *Fusarium avenaceum*. To the best of our knowledge, this is the first report on the inhibition activity of *A. altissima* extracts of young shoots, mature leaves, yellow leaves, petioles of leaves, male inflorescences, bark of 1–2-year branches, bark of tree trunks, and bark of roots against *Fusarium avenaceum*.

### 2.4. TLC–Enzyme Inhibition Activities of 19 Selected Extracts

#### 2.4.1. TLC–α-Glucosidase Inhibitory Activity

**Figure 11:**
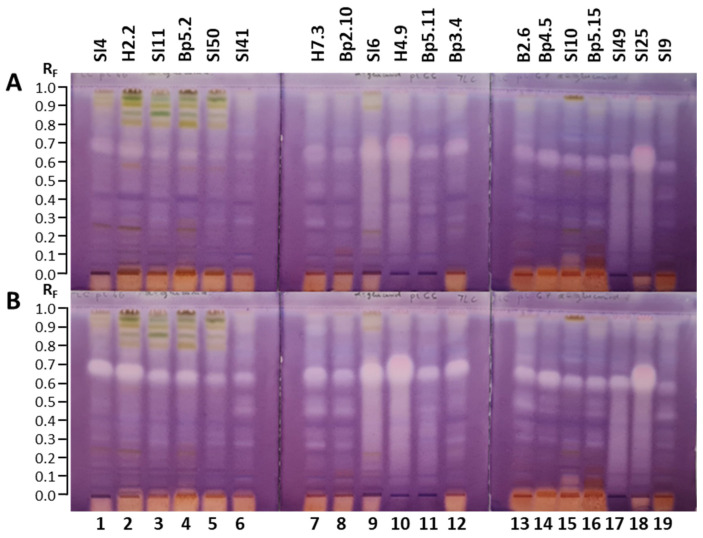
TLC–α-glucosidase inhibition autograms of 19 selected extracts (15 µL, 200 mg/mL) on the TLC silica gel F_254_ plates developed with toluene-isopropyl acetate-methanol (5:4:1, *V/V/V*) and documented under white light instantly (**A**) and 30 min (**B**) after the bioassay. Track 1: young leaves SI4; track 2: young leaves H2.2; track 3: mature leaves SI11; track 4: mature leaves Bp5.2; track 5: yellow leaves SI50; track 6: petioles of leaves SI41; track 7: petioles of leaves H7.3; track 8: young shoots Bp2.10; track 9: bark of 1–2-year branches SI6; track 10: bark of tree trunk H4.9; track 11: bark of roots Bp5.11; track 12: male inflorescences Bp3.4; track 13: petioles of male inflorescences B2.6; track 14: female inflorescences Bp4.5; track 15: young fruits SI10; track 16: petioles of fruits Bp5.15; track 17: bark of tree trunk SI49; track 18: bark of 2-year branches SI25; track 19: mature male inflorescences SI9.

The TLC–α-glucosidase inhibition assay resulted in several bright inhibition zones on a violet background in tracks of all extracts (Figure 11), confirming the inhibitory activity against α-glucosidase in all 19 extracts. The results are summarized in Table 1. For all extracts, bright inhibition zones were far more intense 30 min after the assay (Figure 11B) than instantly after the assay (Figure 11A). More inhibition zones were visible 30 min after the assay (Figure 11B) than instantly (Figure 11A).

Although for all 19 extracts, the most intense inhibition zones were detected in the R_F_ range of 0.58 to 0.77 (Figure 11), the intensity of these zones varied among extracts. The most intense α-glucosidase inhibition zones in this R_F_ range were found at R_F_ ≈ 0.68 for the extract of bark of tree trunk H4.9 (track 10) and at R_F_ ≈ 0.66 for the extract of bark of 2-year branches SI25 (track 18) (Figure 11). However, the upper part of these bright zones appeared to be slightly pink, indicating the presence of two inhibition zones with comparable intensities in cases of both extracts: at R_F_ ≈ 0.65 and R_F_ ≈ 0.73 for the extract of bark of tree trunk H4.9 (Figure 11, track 10) and at R_F_ ≈ 0.63 and R_F_ ≈ 0.70 for the extract of bark of 2-year branches SI25 (Figure 11, track 18). In the same R_F_ range two zones were undoubtedly detected also for the extract of bark of 1–2-year branches SI6 (Figure 11, track 9), but in this case the zone at the lower R_F_ ≈ 0.65 was clearly more intense than the zone at R_F_ ≈ 0.73. As evident from Figure 11, comparable intensities were observed for the zones of extracts of young leaves SI4 (track 1, R_F_ ≈ 0.69) and H2.2 (track 2, R_F_ ≈ 0.67), and extract of male inflorescences Bp3.4 (track 12, R_F_ ≈ 0.67). However, these intensities were slightly lower than those observed for bark of tree trunk H4.9 (track 10), bark of 2-year branches SI25 (track 18), and bark of 1–2-year branches SI6 (track 9) (Figure 11). Similar intensities were detected also for the zones at R_F_ ≈ 0.65 for the extracts of petioles of leaves H7.3 (track 7) and female inflorescences Bp4.5 (track 14) (Figure 11).

Zones with comparable intensities were also obtained at R_F_ ≈ 0.65 for the extracts of bark of roots Bp5.11 (track 11) and mature leaves SI11 (track 3) and Bp5.2 (track 4), and at R_F_ ≈ 0.67 for petioles of male inflorescences B2.6 (track 13) (Figure 11). Compared to the intensity of the zones in the above-mentioned extracts, the intensities of these zones in extracts of bark of roots Bp5.11, petioles of male inflorescences B2.6, mature leaves SI11 and mature leaves Bp5.2 were lower (Figure 11). However, even lower intensities were observed for the zones at R_F_ ≈ 0.65 for young shoots Bp2.10 (track 8) and the zones at R_F_ ≈ 0.63 for the extracts of bark of tree trunk SI49 (track 17), young fruits SI10 (track 15), and petioles of the fruits Bp5.15 (track 16) (Figure 11). The intensities of the zones at R_F_ ≈ 0.64 for the extracts of petioles of leaves SI41 (Figure 11, track 6) and yellow leaves SI50 (Figure 11, track 5) were even lower. Among the 19 extracts, the lowest intensity zone in this R_F_ range was observed at R_F_ ≈ 0.60 for the extract of mature male inflorescences (SI9, Figure 11, track 19).

Other intense α-glucosidase inhibition zones were detected in the R_F_ range of 0.28 to 0.56 for the extracts of bark of tree trunk H4.9 (track 10) and SI49 (track 17), bark of 1–2-year branches SI6 (track 9), and bark of 2-year branches SI25 (track 18) (Figure 11). As shown in Figure 11, additional quite intense zones were detected at R_F_ ≈ 0.28, 0.45 and 0.51 for extracts of petioles of leaves SI41 (track 6) and H7.3 (track 7), as well as for extracts of young shoots Bp2.10 (track 8), petioles of male inflorescences B2.6 (track 13), petioles of fruits Bp5.15 (track 16), and mature male inflorescences SI9 (track 19). Inhibition zones at R_F_ ≈ 0.45 for extracts of petioles of leaves SI41 (track 6) and H7.3 (track 7), and young shoots Bp2.10 (track 8) showed lower intensities than the zones in the R_F_ range between 0.60 and 0.70 (Figure 11). Although the extracts of bark of 1–2-year branches SI6 (track 9), bark of tree trunk H4.9 (track 10), bark of tree trunk SI49 (track 17), and bark of 2-year branches SI25 (track 18) showed intense inhibitory activity in the R_F_ range of 0.25 to 0.60, separate zones cannot be discussed due to tailing. The most intense inhibition zone at R_F_ ≈ 0.28 was associated with the extract of petioles of leaves H7.3 (track 7) (Figure 11). Intense inhibition zones were also observed at R_F_ ≈ 0.90 for extracts of petioles of leaves SI41 (track 6) and H7.3 (track 7), as well as for extracts of young shoots Bp2.10 (track 8), bark of tree trunk H4.9 (track 10), petioles of male inflorescences B2.6 (track 13), petioles of fruits Bp5.15 (track 16), and mature male inflorescences SI9 (track 19) (Figure 11).

**Figure 12:**
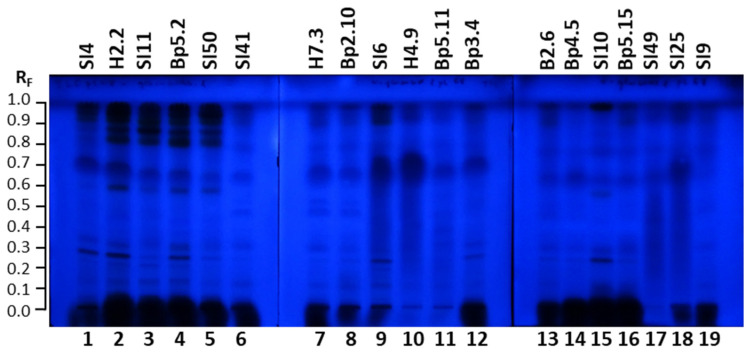
TLC–α-glucosidase inhibition autograms of 19 selected extracts (15 µL, 200 mg/mL) on the TLC silica gel F_254_ plates developed with toluene-isopropyl acetate-methanol (5:4:1, *V/V/V*) and documented at 365 nm 30 min after the assay. Track 1: young leaves SI4; track 2: young leaves H2.2; track 3: mature leaves SI11; track 4: mature leaves Bp5.2; track 5: yellow leaves SI50; track 6: petioles of leaves SI41; track 7: petioles of leaves H7.3; track 8: young shoots Bp2.10; track 9: bark of 1–2-year branches SI6; track 10: bark of tree trunk H4.9; track 11: bark of roots Bp5.11; track 12: male inflorescences Bp3.4; track 13: petioles of male inflorescences B2.6; track 14: female inflorescences Bp4.5; track 15: young fruits SI10; track 16: petioles of fruits Bp5.15; track 17: bark of tree trunk SI49; track 18: bark of 2-year branches SI25; track 19: mature male inflorescences SI9.

The inhibitory activity of all 19 extracts against α-glucosidase was also confirmed with another TLC–α-glucosidase inhibition assay that enabled detection of dark inhibition zones on a blue background in fluorescence mode at 365 nm in tracks of all extracts 30 min after the assay (Figure 12).

In the only report in the literature on the inhibition of α-glucosidase by *A. altissima* extracts/fractions, the extract of bark prepared in water showed much lower inhibition of α-glucosidase than the extract prepared in methanol, whereas the extract prepared in 70% methanol showed significant inhibition of α-glucosidase comparable with that of the positive control [44].

To the best of our knowledge, this is the first report on the inhibition of α-glucosidase by *A. altissima* extracts of young shoots, young leaves, mature leaves, yellow leaves, petioles of leaves, petioles of male inflorescences, petioles of fruits, female inflorescences, male inflorescences, mature male inflorescences, young fruits, bark of 1–2-year branches, bark of 2-year branches, and bark of roots.

#### 2.4.2. TLC–Lipase Inhibitory Activity

**Figure 13:**
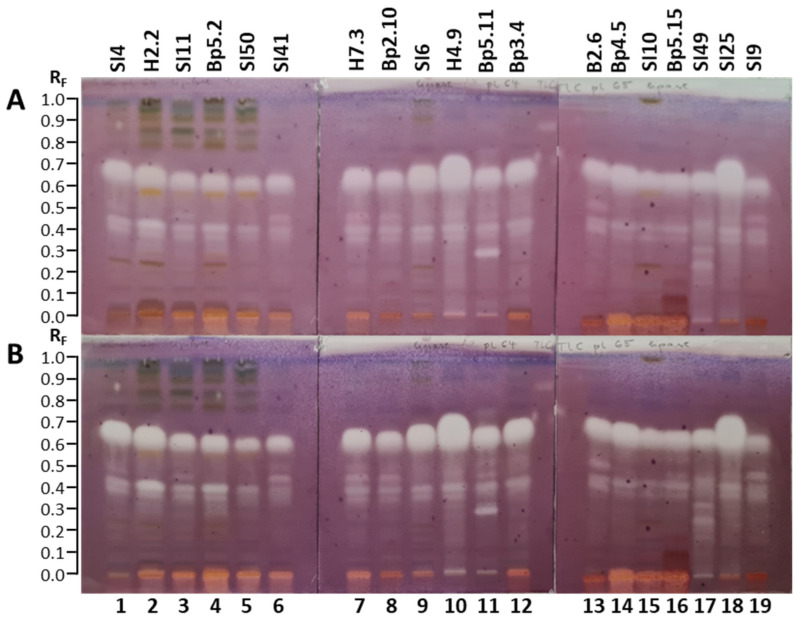
TLC–lipase inhibition autograms of 19 selected extracts (15 µL, 200 mg/mL) on the TLC silica gel F_254_ plates developed with toluene-isopropyl acetate-methanol (5:4:1, *V/V/V*) and documented under white light instantly (**A**) and 30 min after the bioassay (**B**). Track 1: young leaves SI4; track 2: young leaves H2.2; track 3: mature leaves SI11; track 4: mature leaves Bp5.2; track 5: yellow leaves SI50; track 6: petioles of leaves SI41; track 7: petioles of leaves H7.3; track 8: young shoots Bp2.10; track 9: bark of 1–2-year branches SI6; track 10: bark of tree trunk H4.9; track 11: bark of roots Bp5.11; track 12: male inflorescences Bp3.4; track 13: petioles of male inflorescences B2.6; track 14: female inflorescences Bp4.5; track 15: young fruits SI10; track 16: petioles of fruits Bp5.15; track 17: bark of tree trunk SI49; track 18: bark of 2-year branches SI25; track 19: mature male inflorescences SI9.

The TLC–lipase inhibition assay resulted in several bright inhibition zones on a violet background in tracks of all 19 extracts (Figure 13). The results are summarized in Table 1. All inhibition zones were less intense instantly (Figure 13A) than 30 min after the bioassay (Figure 13B). For all extracts, the most intense inhibition zones were detected in the R_F_ range of 0.55 to 0.74 (Figure 13), but the intensity of these zones varied among extracts. The most intense inhibition zone in this R_F_ range was detected for extract of bark of tree trunk H4.9 (track 10, R_F_ ≈ 0.55–0.74), followed by extracts of bark of 2-year branches SI25 (track 18, R_F_ ≈ 0.57–0.74) and bark of 1–2-year branches SI6 (track 9, R_F_ ≈ 0.55–0.69) (Figure 13). As evident in Figure 13, a slightly lower intensity of the zones in this R_F_ range was observed for extracts of young leaves SI4 (track 1, R_F_ ≈ 0.57–0.68), young leaves H2.2 (track 2, R_F_ ≈ 0.56–0.67), petioles of leaves H7.3 (track 7, R_F_ ≈ 0.56–0.67), male inflorescences Bp3.4 (track 12, R_F_ ≈ 0.60–0.71), and female inflorescences Bp4.5 (track 14, R_F_ ≈ 0.59–0.70).

The zones for the extracts of young shoots Bp2.10 (track 8, R_F_ ≈ 0.56–0.66), bark of roots Bp5.11 (track 11, R_F_ ≈ 0.57–0.67), petioles of male inflorescences B2.6 (track 13, R_F_ ≈ 0.60–0.70), young fruits SI10 (track 15, R_F_ ≈ 0.58–0.68), petioles of fruits Bp5.15 (track 16, R_F_ ≈ 0.57–0.67) and bark of tree trunk SI49 (track 17, R_F_ ≈ 0.58–0.68) were slightly less intense compared to the ones already mentioned (Figure 13). Even less intense zones but with comparable intensity and equal R_F_ range, were observed for two pairs of extracts: 1) extracts of mature leaves SI11 (track 3, R_F_ ≈ 0.55–0.64) and Bp5.2 (track 4, R_F_ ≈ 0.55–0.64), and 2) extracts of petioles of leaves SI41 (track 6, R_F_ ≈ 0.55–0.63) and mature male inflorescences SI9 (track 19, R_F_ ≈ 0.55–0.63) (Figure 13). Among all extracts, the lowest intensity was detected for the extract of yellow leaves SI50 (track 5, R_F_ ≈ 0.55–0.62) (Figure 13). For all extracts, most of the other less intense zones were observed in the R_F_ range of 0.28 to 0.48. In this R_F_ range the most intense zones were found at R_F_ 0.40 (Figure 13) for the extracts of young leaves H2.2 (track 2) and mature leaves Bp5.2 (track 4), with slightly higher intensity in the case of young leaves H2.2. Another inhibition zone with a slightly lower intensity than the zones at R_F_ 0.40 in tracks 2 and 4 (Figure 13) was observed at R_F_ 0.29 for the extract of bark of roots Bp5.11 (track 11, Figure 13). As shown in Figure 13, additional inhibition zones were detected at R_F_ 0.26 for the extract of bark of tree trunk SI49 (track 17) and at R_F_ 0.51 for the extracts of bark of roots Bp5.11 (track 11), male inflorescences Bp3.4 (track 12) and petioles of male inflorescences B2.6 (track 13). Lipase inhibition zones were also detected at R_F_ 0.01 for the extract of bark of tree trunk SI49 (track 17) and R_F_ 0.02 for the extracts of bark of the roots Bp5.11 (track 11) and bark of tree trunk SI49 (track 17) (Figure 13).

**Figure A2:**
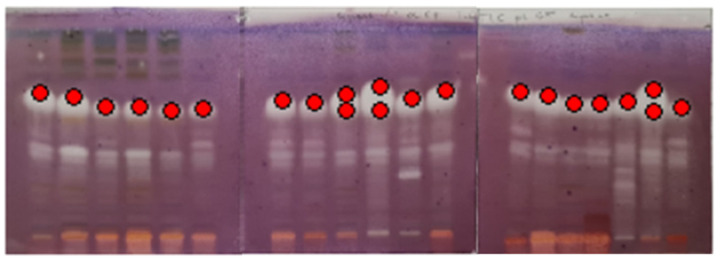
Image analysis of TLC–*lipase* bioautograms of 19 selected extracts (15 µL, 200 mg/mL) on the HPTLC silica gel F_254_ plates developed with toluene-isopropyl acetate-methanol (5:4:1, *V/V/V*) documented under white light. Red circles on the zones were obtained by image analysis with SORBFIL Visualizer TLC Quantitative Evaluation software using the “Tracks/Bright spots” option. Tracks from the left to the right: track 1: young leaves SI4; track 2: young leaves H2.2; track 3: mature leaves SI11; track 4: mature leaves Bp5.2; track 5: yellow leaves SI50; track 6: petioles of leaves SI41; track 7: petioles of leaves H7.3; track 8: young shoots Bp2.10; track 9: bark of 1–2-year branches SI6; track 10: bark of tree trunk H4.9; track 11: bark of roots Bp5.11; track 12: male inflorescences Bp3.4; track 13: petioles of male inflorescences B2.6; track 14: female inflorescences Bp4.5; track 15: young fruits SI10; track 16: petioles of fruits Bp5.15; track 17: bark of tree trunk SI49; track 18: bark of 2-year branches SI25; track 19: mature male inflorescences SI9.

Image analysis performed with SORBFIL Visualizer TLC Quantitative Evaluation software using the “Tracks/Bright spots” option confirmed lipase inhibition zones that were previously detected by visual inspection. However, as shown in Appendix A (Figure A2), within the R_F_ range of 0.55–0.74, image analysis revealed the presence of two distinct inhibition zones in three extracts (Figure A2), whereas visual inspection indicated only one broader zone in the same extracts of bark of 1–2-year branches SI6 (Figure 13B, track 9, R_F_ ≈ 0.55–0.69), bark of tree trunk H4.9 (Figure 13B, track 10, R_F_ ≈ 0.55–0.74), bark of 2-year branches SI25 (Figure 13B, track 18, R_F_ ≈ 0.57–0.74).

There is no report in the literature related to the inhibitory activity of *A. altissima* extracts or fractions against lipase. To the best of our knowledge, this is the first report on the inhibition of lipase by *A. altissima* extracts of young shoots, young leaves, mature leaves, yellow leaves, petioles of leaves, petioles of male inflorescences, petioles of fruits, female inflorescences, male inflorescences, mature male inflorescences, young fruits, bark of 1–2-year branches, bark of 2-year branches, bark of tree trunks, and bark of roots.

#### 2.4.3. TLC–Acetylcholinesterase Inhibition Assay

**Figure 14:**
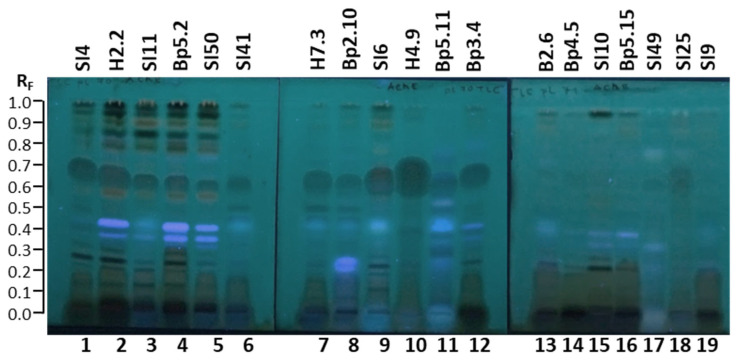
TLC–acetylcholinesterase inhibition autograms of 19 selected extracts (15 µL, 200 mg/mL) on the TLC silica gel F_254_ plates developed with toluene-isopropyl acetate-methanol (5:4:1, *V/V/V*) and documented at 366 nm 20 min after the assay. Track 1: young leaves SI4; track 2: young leaves H2.2; track 3: mature leaves SI11; track 4: mature leaves Bp5.2; track 5: yellow leaves SI50; track 6: petioles of leaves SI41; track 7: petioles of leaves H7.3; track 8: young shoots Bp2.10; track 9: bark of 1–2-year branches SI6; track 10: bark of tree trunk H4.9; track 11: bark of roots Bp5.11; track 12: male inflorescences Bp3.4; track 13: petioles of male inflorescences B2.6; track 14: female inflorescences Bp4.5; track 15: young fruits SI10; track 16: petioles of fruits Bp5.15; track 17: bark of tree trunk SI49; track 18: bark of 2-year branches SI25; track 19: mature male inflorescences SI9.

As shown in Figure 14, the TLC–acetylcholinesterase inhibition assay of 19 extracts resulted in several dark zones on a bluish–greenish fluorescent background, indicating inhibitory activity. The results are summarized in Table 1. The intense blue, fluorescent zones appeared at the same R_F_ and for the same extracts as after development and documentation at 366 nm (before the acetylcholinesterase inhibition assay) (Figure 14). Dark inhibition zones were detected in all 19 extracts 20 min after the assay (Figure 14). As evident from the number and intensities of dark inhibition zones, extracts showed diverse diverse profiles (Figure 14). The broadest dark inhibition zones were detected in the R_F_ range of 0.55 to 0.73 for the following thirteen extracts: young leaves SI4 (track 1), young leaves H2.2 (track 2), mature leaves SI11 (track 3), mature leaves Bp5.2 (track 4), yellow leaves SI50 (track 5), petioles of leaves SI41 (track 6), petioles of leaves H7.3 (track 7), young shoots Bp2.10 (track 8), bark of 1–2-year branches SI6 (track 9), bark of tree trunk H4.9 (track 10), bark of roots Bp5.11 (track 11), and male inflorescences Bp3.4 (track 12) (Figure 14). In the same R_F_ range dark inhibition zones with lower intensity were detected for extracts of petioles of male inflorescences B2.6 (track 13), female inflorescences Bp4.5 (track 14), young fruits SI10 (track 15), petioles of fruits Bp5.15 (track 16), bark of tree trunk SI49 (track 17), and bark of 2-year branches SI25 (track 18) (Figure 14). Among these six extracts, bark of 2-year branches SI25 (track 18) had the most intense zone in the R_F_ range of 0.55 to 0.73 (Figure 14). No dark zone was detected in this R_F_ range for the extract of mature male inflorescences SI9 (track 19) (Figure 14).

Intense dark inhibition zones were also detected at the start positions for the extracts of young leaves SI4 (track 1), young leaves H2.2 (track 2), mature leaves SI11 (track 3), mature leaves Bp5.2 (track 4), yellow leaves SI50 (track 5), petioles of leaves SI41 (track 6), petioles of leaves H7.3 (track 7), young shoots Bp2.10 (track 8), bark of tree trunk H4.9 (track 10), male inflorescences Bp3.4 (track 12), petioles of male inflorescences B2.6 (track 13), female inflorescences Bp4.5 (track 14), young fruits SI10 (track 15), petioles of fruits Bp5.15 (track 16), bark of 2-year branches SI25 (track 18), and mature male inflorescences SI9 (track 19) (Figure 14). The only exceptions were extracts of bark of 1–2-year branches SI6 (track 9), bark of roots Bp5.11 (track 11), and bark of tree trunk SI49 (track 17), in which the dark inhibition zones at the start positions were partly covered (tracks 9 and 11) or completely covered (track 17) by intense blue fluorescent zones (Figure 14).

Sharp, dark inhibition zones were detected at different positions below RF 0.33 for the following extracts: young leaves SI4 (track 1) and H2.2 (track 2), mature leaves Bp5.2 (track 4), bark of 1–2-year branches SI6 (track 9), male inflorescences Bp3.4 (track 12), and young fruits SI10 (track 15) (Figure 14).

Dark inhibition zones were also detected in the R_F_ range of 0.78 to 0.99 in tracks of all 19 extracts (Figure 14). In this R_F_ range, not only the number but also different intensities were observed among the extracts. As shown in Figure 14, the most intense dark zones in this R_F_ range were observed for the extracts of young leaves SI4 (track 1) and H2.2 (track 2), mature leaves SI11 (track 3) and Bp5.2 (track 4), yellow leaves SI50 (track 5), bark of 1–2-year branches SI6 (track 9), and young fruits SI10 (track 15).

Acetylcholinesterase inhibitory activity with IC50 values of 3.28 ± 0.18 μM determined for monoterpenoid chouchunionone A (isolated from the ethanol extract of *A. altissima* leaves) was comparable to that of positive control donepezil (IC50 = 3.12 ± 0.006 µM) [18]. One published study evaluated acetylcholinesterase inhibitory activity using the Ellman method [45] and reported IC50 values of 211.31 ± 1 6.31 µg/mL and 5.3 ± 0.19 µg/mL for *A. altissima* bark extract in 70% methanol and galantamine standard, respectively [16].

To the best of our knowledge, this is the first report on the inhibition of acetylcholinesterase by *A. altissima* extracts of young shoots, young leaves, mature leaves, yellow leaves, petioles of leaves, petioles of male inflorescences, petioles of fruits, female inflorescences, male inflorescences, mature male inflorescences, young fruits, bark of 1–2-year branches, bark of 2-year branches, and bark of roots.

## 3. Materials and Methods

### 3.1. Chemicals

All chemicals used in the study were at least of analytical grade. Ethyl acetate, isopropyl acetate, toluene, dimethyl sulfoxide (DMSO), acetic acid (glacial, 100%), sulfuric acid (95–97%), *p*-methoxybenzaldehyde (anisaldehyde), molybdatophosphoric acid, dipotassium monohydrogen phosphate, and sodium phosphate monobasic dihydrate were obtained from Merck (Darmstadt, Germany). Methanol (LC-MS grade) was obtained from Honeywell (Seelze, Germany), ethanol was obtained from Carlo Erba Reagents (Val-de-Reuil, France), and acetone (≥99.5) was obtained from Sigma-Aldrich (Steinheim, Germany). Primuline (dye content 50%), (2-(4-iodophenyl)-3-(4-nitrophenyl)-5-phenyl-2H-tetrazolium chloride (INT), acetylcholinesterase lyophilizate (from *Electrophorus electricus*, AChE, 200–1.000 U/mg), lipase (from porcine pancreas, ≥125 units/mg), and bovine serum albumin (BSA) were purchased from Sigma-Aldrich (St. Louis, MO, USA), and α-glucosidase (from yeast maltase, 1000 U/mL in 3.2 M ammonium sulphate) was obtained from Megazyme (Bray, Co. Wicklow, Ireland). Fast blue salt B, 3-indoxyl acetate, 2-naphthyl-α-D-glucopyranoside, and α-naphthyl acetate were acquired from Biosynth (Bratislava, Slovakia). MTT (3-[4,5-dimethylthiazol-2-yl]-2,5-diphenyltetrazolium bromide) was obtained from Carl Roth (Karlsruhe, Germany). Magnesium chloride, potassium sulphate, sodium chloride, sodium hydroxide, glycerol, tryptone, glucose and meat extract were obtained from Reanal (Budapest, Hungary). Pancreatic digested gelatin was obtained from Carl Roth (Karlsruhe, Germany). Peptone and yeast extract were obtained from Scharlau (Barcelona, Spain). Sea-salt mix was obtained from Instant Ocean (Blacksburg, VA, USA). Ultrapure water was supplied by a Milli-Q water purification system (18 MΩ cm^−1^) from Millipore (Bedford, MA, USA).

### 3.2. Samples

Plant parts of A. altissima were collected in five localities from four different phytogeographical regions (PGR) of Slovenia (predinnaric PGR: Ljubljana (N 46°4′21,7″ E 14°29′40,79″, 300 m a.s.l.) and Medvode (N 46°08′38.3″ E 14°24′54.1″, 320 m a.s.l.); subpannonian PGR: Pivola by Maribor (N 46°30′28,48″ E 15°37′18,88″, 390 m a.s.l.); alpine PGR: Soča (N 46°20′15.6″ E 13°38′40.7″, 480 m a.s.l.); submediterranean PGR: Strunjan (N 45°32′8,55″ E 13°36′10,17″, 30 m a.s.l.)) and four localities from the Pannonian PGR of Hungary (Leányfalu (N 47°42′11″ E 19°04′25″, 254 m a.s.l.), Harta (N 46°41′44″ E 19°03′13″, 90 m a.s.l.), Balatongyörök (N 46°45′35″ E 17°20′16″, 114 m a.s.l.), and Budapest (N 47°30′50″ E 19°00′43″, 182 m a.s.l.)) (Table 2). The coordinates are in WGS84 (World Geodetic System 1984).

The following plant parts were collected from May to October: young leaves, mature leaves, yellow leaves, petioles of leaves, young shoots, bark of tree trunks, bark of 1–2-year branches, bark of 2-year branches, young fruits, petioles of fruits, male inflorescences, female inflorescences, petioles of male inflorescences, and bark of roots (Table 2). Voucher specimens of Slovene samples are stored in the Herbarium LJU, and voucher specimens of Hungarian samples are stored in the Herbarium of the Plant Protection Institute, CAR, HUN-REN, Budapest, Hungary.

The samples were dried in the air at room temperature and stored in paper bags until they were ground with a Grindomix GM 200 knife mill (Retsch, Haan, Germany) or an SCG 2051BK grinder (Sencor, Říčany, Czech Republic).

### 3.3. Preparation of Extracts

Powdered samples (1 g) were extracted in glass tubes with methanol (5 mL) by vortexing (30 s at 2800 rpm; IKA lab dancer (IKA-Werke, Staufen, Germany)) and ultrasound-assisted extraction (UAE, 15 min at 50 Hz; ultrasonic bath Sonis 3 GT, Iskra Pio d.o.o., Šentjernej, Slovenia) or maceration for 24 h at room temperature. UAE and maceration were followed by centrifugation for 5 min at 4200 rpm (Centric 322 A, Tehtnica, Železniki, Slovenia). Supernatants were filtered through 0.45 µm polyvinylidene fluoride (PVDF) membrane syringe filters (Macherey-Nagel, Dűren, Germany). The obtained filtered supernatants were stored in glass vials at −20 °C. Solutions from vials in which small particles were observed at the bottom after thawing were centrifuged for 5 min at 13,400 rpm (MiniSpin centrifuge, MiniSpin, Eppendorf, Hamburg, Germany). Supernatants (hereafter referred to as extracts) were transferred into GC vials and were used undiluted for TLC and HPTLC analyses.

**Table 2:** Samples (Nos., codes and plant parts), sampling localities in Slovenia (SI) and Hungary (HU), collection dates, and herbarium deposition numbers. Nos. are used for numbering of the extracts below the (HP)TLC plate images in Figure 4, Figure 5, Figure 6, Figure 7, Figure 8, Figure 9, Figure 10, Figure 11, Figure 12, Figure 13 and Figure 14, Figure A1 and Figure A2.

No.	Code	Plant Part	Locality	Collection Date	Herbarium Deposition Number
9	SI6	Bark of 1–2-year branches	SI: Medvode	17 May 2022	LJU10148083
28	SI3	Bark of 1–2-year branches	SI: Soča	15 May 2022	LJU10148084
25	H4.8	Bark of 1–2-year branches	HU: Harta	3 July 2022	PPI-MA-Aa-H4.8
26	B3.8	Bark of 1–2-year branches	HU: Balatongyörök	26 July 2022	PPI-MA-Aa-B3.8
18	SI25	Bark of 2-year branches	SI: Soča	9 August 2022	LJU10148084
21	SI13	Bark of 2-year branches	SI: Ljubljana	3 August 2022	LJU10148087
11	Bp5.11	Bark of roots	HU: Budapest	8 July 2022	PPI-MA-Aa-Bp5.11
22	SI16	Bark of tree trunk	SI: Ljubljana	3 August 2022	LJU10148087
17	SI49	Bark of tree trunk	SI: Strunjan	7 October 2022	LJU10148086
10	H4.9	Bark of tree trunk	HU: Harta	3 July 2022	PPI-MA-Aa-H4.9
14	Bp4.5	Female inflorescences	HU: Budapest	14 June 2022	PPI-MA-Aa-Bp4.5
12	Bp3.4	Male inflorescences	HU: Budapest	8 June 2022	PPI-MA-Aa-Bp3.4
3	SI11	Mature leaves	SI: Ljubljana	3 August 2022	LJU10148087
4	Bp5.2	Mature leaves	HU: Budapest	8 July 2022	PPI-MA-Aa-Bp5.2
23	H5.2	Mature leaves	HU: Harta	13 August 2022	PPI-MA-Aa-H5.2
19	SI9	Mature male inflorescences	SI: Ljubljana	21 June 2022	LJU10148087
6	SI41	Petioles of leaves	SI: Ljubljana	6 October 2022	LJU10148087
24	H5.3	Petioles of leaves	HU: Harta	13 August 2022	PPI-MA-Aa-H5.3
7	H7.3	Petioles of leaves	HU: Harta	24 October 2022	PPI-MA-Aa-H7.3
20	SI12	Petioles of leaves and rachises	SI: Ljubljana	3 August 2022	LJU10148087
13	B2.6	Petioles of male inflorescences	HU: Balatongyörök	7 June 2022	PPI-MA-Aa-B2.6
16	Bp5.15	Petioles of fruits	HU: Budapest	8 July 2022	PPI-MA-Aa-Bp5.15
5	SI50	Yellow leaves	SI: Pivola	7 October 2022	LJU10148085
15	SI10	Young fruits	SI: Ljubljana	21 June 2022	LJU10148087
1	SI4	Young leaves	SI: Medvode	17 May 2022	LJU10148083
2	H2.2	Young leaves	HU: Harta	16 May 2022	PPI-MA-Aa-H2.2
8	Bp2.10	Young shoots	HU: Budapest	13 May 2022	PPI-MA-Aa-Bp2.10
27	SI2	Young shoots	SI: Soča	15 May 2022	LJU10148084

### 3.4. Preparation of Detection Reagents

All detection reagents, including 10% (*V/V*) sulfuric acid in ethanol [46] (p. 402), were prepared for dipping. Anisaldehyde detection reagent was prepared by mixing glacial acetic acid (20 mL) and methanol (170 mL). During cooling with cold water, 16 mL of sulfuric acid was added in a dropwise manner, and subsequently, anisaldehyde (1 mL) was added to the mixture [46] (p. 195). Molybdatophosphoric acid detection reagent was prepared by dissolving 20 g of molybdatophosphoric acid in 200 mL of ethanol [46] (p. 342). Primuline detection reagent was prepared from 5 mg of primuline and 100 mL of acetone-water (4:1, *V/V*) mixture [47].

### 3.5. TLC and HPTLC Analyses

Analyses were performed on TLC silica gel 60 F_254_ aluminum sheets (20 cm × 20 cm—cut into 20 cm × 10 cm pieces, Art. No. 1.05554, Merck), HPTLC silica gel 60 F_254_ aluminum sheets (20 cm × 20 cm—cut into 20 cm × 10 cm pieces, Art. No. 1.05548, Merck), HPTLC silica gel 60 F_254_ glass-backed plates (20 cm × 10 cm, Art. No. 1.05642, Merck), and HPTLC silica gel 60 glass-backed plates (20 cm × 10 cm, Art. No. 1.05641, Merck).

Application of extracts was performed by means of an automatic TLC Sampler 4 (CAMAG, Muttenz, Switzerland) or Autosampler 3 (CAMAG). Extracts were applied on the plates as 8 mm bands, 8 mm from the bottom of the plate and 20 mm from the left edge. For each particular analysis/assay, all extracts were applied on the plates in equal volumes. Application volumes were 10 µL for analyses that included post-chromatographic derivatization with sulfuric acid reagent, anisaldehyde reagent, molybdatophosphoric acid reagent, and primuline reagent. Application volumes for the nine TLC and HPTLC antibacterial bioassays, antifungal bioassays and enzyme inhibition assays are described in Section 3.6. The plates were developed up to 9 cm in a saturated (10 min without filter paper) twin-trough chamber (CAMAG) for 20 cm × 10 cm plates using 10 mL of the developing solvent toluene-isopropyl acetate-methanol (5:4:1, *V/V/V*) poured only in one trough. The developing time was 28 min. The developed plates were dried (40 min) in a stream of cold air (a hair dryer placed on a holder).

Post-chromatographic derivatization was performed using one of the four detection reagents prepared as described in Section 3.4. A Chromatogram immersion device III (CAMAG) was applied for dipping the plates into detection reagents. Dipping the plates for 2 s into sulfuric acid reagent, anisaldehyde reagent or molybdatophosphoric acid reagent was followed by drying the plates in a stream of warm air (a hair dryer) and heating on a TLC plate heater (CAMAG) at 110 °C (5 min), 110 °C (2 min), or 150 °C (35 min), respectively.

Documentation of the images of the plates was performed after development and after post-chromatographic derivatization by means of a DigiStore 2 documentation system (CAMAG) controlled by winCATS software (CAMAG; Version 1.4.9.2001), TLC Visualizer controlled by visionCATS 3.1 software (CAMAG; Version 3.1.21109.3) or a Cybershot DSC-HX60 camera (Sony, Neu-Isenburg, Germany) used with or without a UV lamp (CAMAG). Images of all developed plates were captured under white light (Vis) and at 254 nm (UV) and 366 nm (FLD). Illumination conditions used for capturing images of the plates after post-chromatographic derivatization differed depending on the detection reagent. The conditions were as follows: white light and 366 nm for sulfuric acid and anisaldehyde detection reagent; white light for molybdatophosphoric acid detection reagent; 366 nm for primuline detection reagent. Illumination conditions used for capturing images of the plates after TLC and HPTLC–antimicrobial assays, and TLC–enzyme inhibition assays are described for each assay in Section 3.6.

Densitometry was performed with slit-scanning densitometer TLC Scanner 3 (CAMAG) set in the absorption/reflectance mode at 535 nm after derivatization with anisaldehyde reagent. The slit length and width were 5.0 mm and 0.3 mm, respectively. The scanning speed was 20 mm/s.

Image analysis of the plate images that were not documented by the CAMAG system were performed by SORBFIL Visualizer TLC Quantitative Evaluation software (Version 2.4.0.3000, Sorbpolymer, Krasnodar, Russia). Figure A1 and Figure A2 in Appendix A were obtained using snipping, as export from the program was not possible.

### 3.6. TLC and HPTLC–Effect-Directed Analysis (EDA) with Antibacterial, Antifungal and Enzyme Inhibition Assays

TLC and HPTLC–effect-directed analyses were performed for 19 selected extracts. For each particular assay (antibacterial, antifungal and enzyme inhibition assays), all 19 extracts were applied on the plates in equal volumes. Application volumes for TLC and HPTLC–antimicrobial bioassays were as follows: 5 µL for the *Aliivibrio fischeri* bioassay; 10 µL for the *Bacillus subtilis* bioassay, the *Rhodococcus fascians* bioassay, and the *Pseudomonas syringae* pv. *maculicola* bioassay; and 20 µL for the *Bipolaris sorokiniana* bioassay and the *Fusarium avenaceum* bioassay. Application volumes for all TLC–enzyme inhibition assays (α-glucosidase, lipase, and acetylcholinesterase) were 15 µL. TLC and HPTLC plates were developed as described in Section 3.5.

Incubations of the TLC and HPTLC plates were performed using a Heratherm™ IMC18 Compact Microbiological Incubator (Thermo Fisher Scientific, Waltham, MA, USA) or Model FD 56 drying and heating chamber with forced convection (BINDER, Tuttlingen, Germany). The plates were incubated in closed, humid boxes. Paper towels soaked with water were placed on the bottom of each box. Carriers such as plastic boxes, Petri dishes, etc., were placed on the paper towels. The plates were then horizontally placed on the carriers, on top of the stack. Documentation at 254 nm and 366 nm and under white light, as well as image analysis were performed as described in Section 3.5.

#### 3.6.1. TLC–*Aliivibrio fischeri* Bioassay

*Aliivibrio fischeri* was grown in ocean medium (tryptone (5 g/L), yeast extract (3 g/L), glycerol (3 g/L), sea salt mix (35 g/L), and concentrated sodium hydroxide solution to set the pH around 7.2) at 28 °C by shaking at 120 rpm to reach an optical density of 2.3–2.6, measured at 600 nm (OD_600_) [41]. The developed and dried TLC plates were dipped into *Aliivibrio fischeri* cell suspension for 8 s. The plates were then placed on a glass plate with a frame, covered by another glass plate to retain the moisture, and bioluminescent bioautograms were instantly recorded by a cooled CCD camera of the iBright FL1500 Imaging System (Thermo Fisher Scientific, Waltham, MA, USA) using exposition times of 40–80 s. Documentation of the bioautograms was repeated after 60 min. The zones with antibacterial activity were detected as dark zones on a bright bioluminescent background.

#### 3.6.2. TLC–*Bacillus subtilis* Bioassay

*Bacillus subtilis* was grown in lysogeny broth (tryptone (10 g/L), yeast extract (5 g/L) and sodium chloride (10 g/L)) at 37 °C by shaking at 120 rpm to reach the late exponential phase (5 × 10^8^ cells/mL, OD_600_ = 1.2) [41]. The developed and dried TLC plates were dipped into *Bacillus subtilis* cell suspension for 8 s. After unglazing in a vertical position, the wet plates were incubated for 2 h at 37 °C in a humid box. This was followed by dipping the plates into an aqueous solution of MTT (3-[4,5-dimethylthiazol-2-yl]-2,5-diphenyltetrazolium bromide; 1 mg/mL) vital dye and incubation for 30 min at 37 °C in a humid box. Images of the plates were documented instantly after the bioassay (t = 0 min) or later (t > 0 min) under white light (reflectance mode). Antibacterial activity was detected as bright zones on a bluish background.

#### 3.6.3. TLC–*Rhodococcus fascians* Bioassay

*Rhodococcus fascians* was grown in Waksman broth (peptone (5 g/L), meat extract (5 g/L), sodium chloride (5 g/L), and glucose (10 g/L); saturated sodium hydroxide solution to set the pH around 7.4) at 30 °C by shaking at 120 rpm to reach the late exponential phase (5 × 10^8^ cells/mL, OD_600_ = 1.2) [37]. The developed and dried TLC plates were dipped into *Rhodococcus fascians* cell suspension for 8 s. After unglazing in a vertical position, the wet plates were incubated for 2 h at 30 °C in a humid box. This was followed by dipping the plates into an aqueous solution of MTT (1 mg/mL) vital dye and incubation in a humid box for 30 min at 30 °C. The images of the plates were documented instantly after bioassay (t = 0 min) or later (t > 0 min) under white light (reflectance mode). Antibacterial activity was detected as bright zones on a bluish background.

#### 3.6.4. HPTLC–*Pseudomonas syringae* pv. *maculicola* Bioassay

Luminescent Arabidopsis pathogen *Pseudomonas syringae* pv. *maculicola* chromosomally tagged with lux-CDABE genes was provided by Jun Fan from the John Innes Center, Department of Disease and Stress Biology (Norwich, UK). *Pseudomonas syringae* pv. *maculicola* was grown in gelatin broth (pancreatic digested gelatin (20 g/L), potassium sulphate (10 g/L), magnesium chloride (1.4 g/L), and glycerol (10 mL/L)) at 28 °C by shaking at 120 rpm to reach the late exponential phase (5 × 10^8^ cells/mL, OD_600_ = 1.2) [40]. The developed and dried HPTLC plates were dipped into *Pseudomonas syringae* pv. *maculicola* cell suspension for 8 s. The plates were then placed on a glass plate with a frame, covered by another glass plate, and bioluminescent bioautograms were recorded by means of an iBright FL1500 Imaging System (Thermo Fisher Scientific) using exposition times of 40–80 s. Zones with antibacterial activity were detected as dark zones on a bright, bioluminescent background.

#### 3.6.5. HPTLC–*Bipolaris sorokiniana* Bioassay

*Bipolaris sorokiniana* (*Sacc*.) Shoemaker H-299 (NCBI GenBank accession No. MH697869) was collected in Hungary. *Bipolaris sorokiniana* was grown in lysogeny broth (see Section 3.6.2) by shaking at 120 rpm at 21 °C for 3 days. Washed mycelium in lysogeny broth was cut into small pieces with a FastPrep-24 Classic homogenizer (7 pieces of 2 mm glass beads in a 2 mL Eppendorf tube at 4.5 m/s twice for 20 s; MP Bio, Beograd, Serbia) [40]. The developed and dried HPTLC plates were dipped into *Bipolaris sorokiniana* mycelium suspension diluted to OD_600_ 0.4 for 8 s. After unglazing in a vertical position, the wet plates were incubated for 48–72 h at 21 °C in a humid box. Images of the plates were documented under white light (reflectance mode). Antifungal activity was detected as zones with a lack of visible dark gray *Bipolaris sorokiniana* hyphae.

#### 3.6.6. HPTLC–*Fusarium avenaceum* Bioassay

*Fusarium avenaceum* strain IMI 319947 was obtained from the CABI-IMI Culture Collection (Egham, UK). *Fusarium avenaceum* was grown in lysogeny broth (see Section 3.6.2) by shaking at 120 rpm at 21 °C for 3 days. Washed mycelium in lysogeny broth was cut into small pieces with a FastPrep-24 Classic homogenizer (7 pieces of 2 mm glass beads in a 2 mL Eppendorf tube at 4.5 m/s twice for 20 s; MP Bio, Beograd, Serbia) [40]. The developed and dried HPTLC plates were dipped into *Fusarium avenaceum* mycelium suspension diluted to OD_600_ 0.4 for 8 s. After unglazing in a vertical position, the wet plates were incubated for 48–72 h at 21 °C in a humid box. After 72 h, the bioautograms were stained by spraying 2-(4-iodophenyl)-3-(4-nitrophenyl)-5-phenyl-2H-tetrazolium chloride (INT, 1 mg/mL in water). Images of the plates were documented under white light (reflectance mode). Antifungal activity was detected as zones with a lack of visible *Fusarium avenaceum* hyphae.

#### 3.6.7. TLC–α-Glucosidase Inhibition Assays

Two TLC–α-glucosidase inhibition assays were performed. For the first assay, a substrate solution was prepared by dissolving 2-naphthyl-α-D-glucopyranoside (10 mg) substrate in DMSO (0.4 mL) and diluting the resulting solution with phosphate buffer (9.6 mL, 0.1 M, pH 7.5). The developed and dried TLC plate was sprayed with a substrate solution. This was followed by spraying the plate with α-glucosidase solution (5 U/mL) prepared in phosphate buffer (0.1 M, pH 7.5) [39]. After unglazing, the wet plate was incubated for 20 min at 37 °C in a humid box. This was followed by spraying the plate with chromogenic reagent fast blue salt B (1 mg/mL in water) and drying the plate. Images of the plate were documented instantly after the bioassay (documentation time (t) = 0 min) or later (t > 0 min) under white light (reflectance mode). Inhibition zones were detected as bright zones on a violet background.

For the second assay, a 4-methylumbelliferyl-α-D-glucopyranoside substrate was dissolved in DMSO (10 mg in 0.4 mL) and diluted with 9.6 mL phosphate buffer (0.1 M, pH 7.5). The developed and dried chromatograms were first sprayed (airbrush, Revell, Bünde, Germany) with the substrate solution and then with α-glucosidase solution (5 units mL^−1^ in 0.1 M phosphate buffer, pH 7.5). After spraying with each solution, the layers were allowed to become unglazed. Then the bioautograms were incubated at 37 °C, at 100% humidity. After 60 min of incubation, the bioautograms were documented under a UV lamp (CAMAG) at 365 nm. Dark zones indicate inhibition against the blue fluorescent background (active enzyme reveal blue fluorescent 4-methylumbelliferol from the substrate) [39].

#### 3.6.8. TLC–Lipase Inhibition Assay

The developed and dried TLC or HPTLC plate was dipped into a substrate solution containing α-naphthyl acetate (1 mg/mL) in ethanol for 4 s. This was followed by drying in a stream of cold air (a hair dryer), followed by dipping the plate for 4 s into lipase solution (0.8 mg/mL) containing bovine serum albumin (BSA, 1 mg/mL), which was prepared in phosphate buffer (0.1 M, pH 7.5) [39]. After unglazing, the plate was incubated for 15 min at 37 °C in a humid box. This was followed by spraying the plate with chromogenic reagent fast blue salt B (1 mg/mL in water) and drying. Images of the plate were documented instantly after the bioassay (t = 0 min) or later (t > 0 min) under white light (reflectance mode). Inhibition zones were detected as bright zones on a violet background.

#### 3.6.9. TLC–Acetylcholinesterase Inhibition Assay

The developed and dried TLC plate was dipped for 4 s into a substrate solution prepared by dissolving 3-indoxyl acetate (25 mg) in DMSO (2 mL) and diluting the obtained solution with phosphate buffer (48 mL, 0.1 M, pH 7.5). Dipping was followed by spraying the plate with acetylcholinesterase solution (AChE, 5 U/mL) that also contained BSA (1 mg/mL) prepared in phosphate buffer (0.1 M, pH 7.5) [38]. After unglazing, the plate was incubated for 20 min at 37 °C in a humid box and documented immediately at 366 nm. Inhibition zones were detected as dark zones on a bluish–greenish fluorescent background.

## 4. Conclusions

This is the first study to evaluate crude extracts prepared from different parts of *A. altissima* using hyphenation of chromatography (with and without chemical derivatization) and *in vitro* antimicrobial activity and enzyme inhibitor activity assays. A combination of high-performance thin-layer chromatography with a set of four antibacterial assays, two antifungal assays and three enzyme inhibitor assays showed to be a really powerful tool, resulting in fast and cost-effective screening focused on the discovery of the potential of *A. altissima* (samples from Slovenia and Hungary) as a source of compounds with antimicrobial and enzyme inhibitor activities. In other words, due to the separation performed, combining chromatographic profiles (obtained before and after derivatization) and bioactivity profiles after visual inspection or image analysis provided more information than can be provided by disk diffusion tests or spectrophotometric methods often used in *in vitro* bioassays.

Bioactivity profiles that included antimicrobial (antibacterial and antifungal) and enzyme inhibitor activities were obtained for 19 crude extracts prepared from the following *A. altissima* parts: young shoots, young leaves, mature leaves, yellow leaves, petioles of leaves, petioles of male inflorescences, petioles of fruits, female inflorescences, male inflorescences, mature male inflorescences, bark of 1–2-year branches, bark of 2-year branches, bark of tree trunks, and bark of roots. These bioactivity profiles showed that all 19 crude extracts possessed the following activities: (1) antibacterial activities against Gram-positive bacteria (*Bacillus subtilis*, *Rhodococcus fascians*) and Gram-negative bacteria (*Aliivibrio fischeri*, *Pseudomonas syringae* pv. *maculicola*) and (2) inhibitory activities against enzymes α-glucosidase, lipase, and acetylcholinesterase. To the best of our knowledge, this is the first report on the antibacterial activities of tree of heaven extracts: (1) extracts of all studied parts against *Rhodococcus fascians*, *Aliivibrio fischeri* and *Pseudomonas syringae* pv. *maculicola* and (2) extracts of all studied parts except leaves, bark of young branches and bark of tree trunks against *Bacillus subtilis*. This is also the first report on the activities of tree of heaven extracts against two fungal plant pathogens: (1) *Bipolaris sorokiniana*—extracts of young shoots, young leaves, mature leaves, petioles of leaves, and bark of roots; and (2) *Fusarium avenaceum*—extracts of young shoots, mature leaves, yellow leaves, petioles of leaves, male inflorescences, bark of 1–2-year branches, bark of tree trunks, and bark of roots. To the best of our knowledge, this is also the first report on the inhibitory activities of tree of heaven extracts: (1) extracts of all studied parts except bark of tree trunks against α-glucosidase, (2) extracts of all studied parts against lipase, and (3) extracts of all studied parts except bark of tree trunks against acetylcholinesterase.

This study confirmed that extracts from different plant parts of tree of heaven possess multiple bioactivities, highlighting the promising potential for utilizing this alien invasive plant species for various beneficial applications. Future research can build on these findings by identifying individual bioactive compounds, testing their activities beyond *in vitro* assays, and exploring large-scale utilization strategies. The results of this study can initiate further evaluation of the potential large-scale applicability of the biomass from tree of heaven—an invasive alien plant species—for isolation of active compounds, which could result in the protection of the environment, new products and other economic benefits. All the suggested future activities can contribute to mitigation of the problems that this invasive alien plant species presents to Slovenia and Hungary, as well as other countries.

## Data Availability

The original contributions presented in this study are included in the article. Further inquiries can be directed to the corresponding authors.
